# Spontaneous Helical Alignment of Smooth Muscle Cells to Form a Medial Layer for Engineered Microvasculature

**DOI:** 10.1002/adhm.202505938

**Published:** 2026-06-29

**Authors:** Victoria D. Vest, Katherine J. Young, Isabella K. Holtz, Mark Mc Veigh, James D. West, Leon M. Bellan

**Affiliations:** ^1^ Department of Mechanical Engineering Vanderbilt University Nashville Tennessee USA; ^2^ Department of Biomedical Engineering Vanderbilt University Nashville Tennessee USA; ^3^ Department of Medicine, Health, and Society Vanderbilt University Nashville Tennessee USA; ^4^ Interdisciplinary Materials Science Program Vanderbilt University Nashville Tennessee USA; ^5^ School of Medicine Vanderbilt University Nashville Tennessee USA

**Keywords:** engineered microvasculature, resistance vessels, tissue engineering

## Abstract

Microscale small resistance vessels (SRVs) are key regulators of local vascular resistance and tissue perfusion, yet, compared to efforts toward engineering capillaries (“exchange vessels”) and vascular grafts (“conduit vessels”), there has been limited effort devoted to engineering SRVs with appropriate architecture and function. Natural SRVs rely on a medial layer of helically arranged contractile smooth muscle cells (SMCs) to modulate lumen diameter, thereby regulating local fluidic resistance. However, current SRV‐scale engineered vasculature either lacks an SMC layer or contains unaligned or non‐contractile SMCs, rendering such constructs incapable of recapitulating the essential function of natural SRVs. Here, we demonstrate that appropriate choice of fabrication parameters can promote spontaneous helical alignment of SMCs seeded on the walls of SRV‐sized channels within a hydrogel (without any patterning of topography or binding sites). The resulting alignment angle is sensitive to the composition of extracellular matrix proteins coating the attachment surface, SMC seeding density, and channel diameter. SMCs within these constructs exhibit functional and morphological hallmarks of a contractile phenotype, including biochemical response to vasoconstrictor endothelin‐1 (ET‐1). Establishing an aligned, contractile phenotype SMC layer represents a critical step toward engineering SRVs with vasoreactive functionality mimicking that of natural vessels.

## Graphical abstract

Spontaneous helical alignment of smooth muscle cells is induced within resistance‐vessel‐sized channels patterned within a hydrogel. The extent of the cells’ orientation angle is dependent on the presence and composition of ECM proteins lining the channel wall and cell seeding density. This helical alignment is a key step toward engineering microscale resistance vessels with vasoreactive functionality.

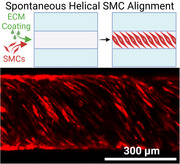

## 1 Introduction

Resistance vessels—arterioles (resting lumen diameter roughly 10–50 µm) and small resistance arteries (50–500 µm)—play a critical role in the cardiovascular system by providing ∼80% of vascular resistance and modulating tissue perfusion by altering their lumen diameters in response to various stimuli [1, 2]. Dysfunction of these vessels is implicated in multiple diseases and conditions, including cardiovascular diseases [3, 4, 5], pulmonary hypertension [6, 7, 8], diabetes [9, 10, 11], chronic kidney disease [12, 13, 14], and Alzheimer's disease [15, 16, 17]. Despite the clear importance of small resistance vessels (SRVs) in normal tissue function, as well as their potential involvement in a wide array of pathologies, little progress has been made toward replicating their structure and function in engineered tissue structures, even while significant progress has been made toward engineering large‐diameter implantable vascular grafts [18, 19] (functioning as conduit vessels) and capillary‐like microvasculature (functioning as exchange vessels) [20, 21]. In vitro tissue models that incorporate engineered SRVs with structure and function matching those of natural vessels have the potential to significantly advance our understanding of disease pathobiology and enhance drug discovery efforts. Moreover, the incorporation of vasoreactive SRVs into implantable engineered tissue constructs would enhance their utility by enabling stimulus‐responsive modulation of tissue perfusion (similar to that seen in the natural microcirculation), representing a critical step toward meaningful replacement of functional deficits.

**FIGURE 1:**
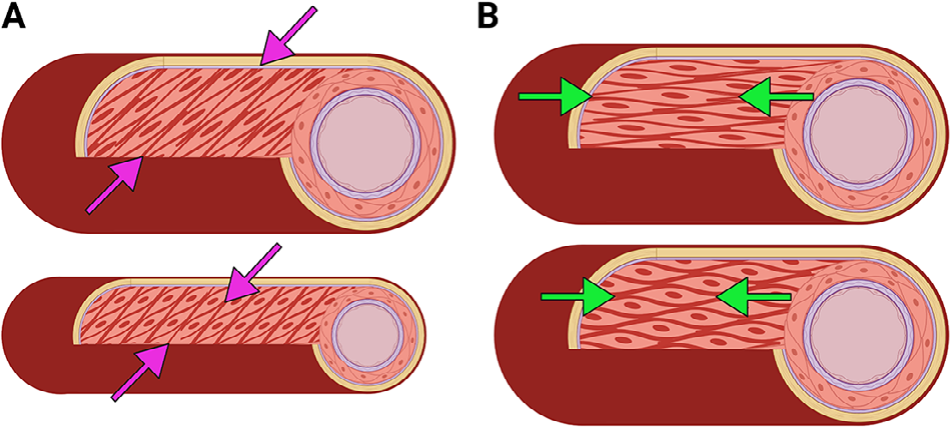
Schematic illustrating the importance of SMC orientation for medial layer functionality. Contraction of SMCs oriented at a bias to the channel axis (purple arrows) has the potential to cause constriction or dilation (A), while contraction of SMCs parallel to the channel axis (green arrows) would not affect lumen diameter (B).

Most natural blood vessels (excluding capillaries) have walls comprising three coaxial cell layers: intima, media, and adventitia. The intima—a monolayer of endothelial cells (ECs)—provides a physical barrier between blood and the surrounding tissue, regulates vessel permeability, and senses and transduces mechanical forces imparted by blood flow. The intima is seated on a thin layer of elastic fibers—the internal elastic lamina (IEL)—which physically separates it from the media. The medial layer is composed of tightly packed smooth muscle cells (SMCs) arranged helically (sometimes called “circumferentially”) around the vessel. This helical arrangement allows the SMCs to regulate vessel diameter by collectively contracting or relaxing in response to various mechanical and biochemical cues (Figure 1), thereby inducing constriction or dilation of the lumen. The adventitia is composed mainly of collagen bundles, nerve endings, and sparse embedded fibroblasts, providing structural support for the vessel. Variation in the wall structure between vessel types is related to the function of the various vessels. Notably, capillaries lack this trilayer architecture and have only an EC monolayer wall, which allows exchange between the blood and the surrounding tissue. For SRVs, the medial layer of SMCs, helically arranged and capable of mechanical actuation, is critical to their primary function, which is to modulate fluidic resistance and thereby regulate local blood flow. The helical alignment of the SMCs in the medial layer is necessary to couple cell contraction/relaxation to vessel constriction/dilation (Figure 1).

Over the past several decades, there has been extensive work focused on engineering perfusable microvasculature capable of supporting the viability of cells cultured in thick tissue constructs [22]. Fabrication techniques closely mimicking natural vasculogenesis and angiogenic sprouting processes have been employed to produce architecture closely resembling natural capillary beds [23, 24, 25], while “top‐down” fabrication techniques such as sacrificial patterning [26, 27] and bioprinting [28, 29, 30] have been used to form channel networks of various geometries [31, 32, 33, 34, 35]. While impressive work has been done toward patterning microvessels to mimic vascular networks, in all the work described above the vessels are lined with a single layer of endothelial cells and therefore serve as exchange vessels—delivering oxygen and nutrients to cells and carrying away waste products. While this is a vital function in both engineered tissues and in vivo, other vascular functions—particularly the vasoreactivity exhibited by resistance vessels—have been largely neglected in engineered microvasculature. In particular, many prior studies demonstrate the production of engineered vessels with lumen diameters at the resistance vessel scale but then provide these vessels only with exchange vessel architecture and functionality. To address this mismatch and impart resistance vessel functionality (vasoreactivity) to resistance vessel‐scale engineered vessels, a properly organized medial layer must be produced.

Prior efforts toward creating SMC layers in engineered vasculature have primarily focused on generating vascular grafts on a millimeter scale, employing a variety of approaches. Two common techniques rely on creating a cylindrical mold for cell seeding [36, 37, 38] or forming 2D cell sheets and rolling them around a tube [39, 40]. These methods in general do not translate well to the microscale. More recently, coaxial extrusion bioprinting methods have been used to produce vessels with SMCs embedded throughout the vessel wall, both in mm‐scale and smaller vessels with lumen diameters down to ∼200 µm [41, 42, 43, 44, 45, 46]. However, the SMCs in these prior studies were not helically aligned, and additionally resided in a “medial layer” notably thicker (∼10×) than that found in natural resistance vessels (which typically have medial layers consisting of only a few layers of SMCs). Such natural geometries have yet to be demonstrated with current extrusion printing technology. Most importantly, extrusion methods generally produce medial layers with SMCs dispersed throughout the layer and oriented randomly with respect to the channel axis. Other methods are necessary to create engineered SRVs in in vitro models that exhibit medial layer organization similar to native vessels. In particular, one promising alternative approach is to culture layers of SMCs on the interior wall of channels patterned in a hydrogel. In their recent work, Zhu et al. have produced SRV‐scale vasculature with both medial layer and endothelium [47] capable of passive diameter changes in response to periodic elevated pressure provided by pulsatile flow. However, the presence of SMCs is not sufficient to recapitulate the vasoreactive functionality of the medial layer of resistance vessels in vivo; it is also crucial that the SMCs are both aligned helically around the vessel and capable of appropriate response to vasoactive stimuli.

While extrusion bioprinting methods can be used effectively to induce cell alignment parallel to the extruded channel axis [48], they have not yet produced helically or perpendicularly aligned SMC layers, and indeed most commonly incorporate unaligned SMCs embedded in 3D throughout thick walls. However, other fabrication methods have been used to induce helical or perpendicular alignment of SMCs in a layer. A common technique is patterning a 2D surface with either topographical [49, 50] or biochemical [51, 52] cues and then seeding the cells onto the patterned surface. 2D‐patterned surfaces can be rolled up, along with preferentially oriented SMCs, to create a tubular structure [53, 54]. Recently, a method for self‐rolling cell layers was used to produce freestanding microvessels with a multilayered wall architecture and (employing lithographically patterned binding sites) helically oriented SMCS [55] sustained under static culture conditions. Topographic patterning approaches, including the use of wrinkled PDMS [56] or gelatin microribbons wrapped around a small removable needle [57], have also been used to produce helically oriented SMCs in a microchannel. While the results achieved by these approaches are impressive, it is unclear whether they would eventually translate to fabrication of smaller vessels or a more complex branching network of vessels. An approach that avoids the use of painstaking lithographic or topographical patterning of guiding features and instead relies on spontaneous organization of the cells due to biochemical cues presented throughout the lumen wall could enable more robust resistance vessel biofabrication. To date, however, no studies have demonstrated techniques to produce perfusable, SRV‐sized channels that contain a medial layer of helically aligned SMCs through a hydrogel.

In addition to architectural organization, phenotypic regulation is vital to controlling SMC behavior and function in resistance vessels [58, 59, 60, 61]. Synthetic and contractile SMCs sit at either end of a spectrum of intermediate phenotypes, which allow blood vessels to respond as needed in a variety of scenarios. Synthetic SMCs have a compact “rhomboid” morphology and are highly proliferative and migratory, while contractile SMCs are long and spindle‐shaped, proliferate and migrate very little, and have many contractile filaments. As vessels mature, the SMC population shifts from mainly synthetic to highly contractile. In vivo, the phenotype and associated behavior of SMCs in the vascular wall are regulated by a complex interplay of genetic programming with topographical and biochemical cues that are still not fully understood. Studies have indicated effects of extracellular matrix (ECM) composition and organization [62, 63, 64, 65], matrix compliance [66, 67, 68], soluble signaling factors [69, 70, 71, 72], cell‐to‐cell signaling [73, 74], and the mechanical forces associated with pulsatile flow [75, 76, 77], on SMC phenotypic regulation. There is ambiguity as to the effects of each of these, and there seems to be significant interplay between these various factors [78, 79]. The challenge of engineering functional, biomimetic resistance vessels with contractile function similar to that of native vessels demands careful consideration of these factors and their impact on SMC phenotype.

Currently, engineering SRV‐scale microvasculature with appropriate trilayer microarchitecture as well as proper cell phenotype and function—particularly of the medial layer of helically aligned SMCs—remains a significant unmet challenge. In this study, we first explore key aspects of the biochemical microenvironment to determine conditions that favor SMC adhesion and contractile phenotype. The results of these studies enable us to produce a layer of SMCs that exhibit spontaneous helical alignment, without the need to spatially pattern alignment cues (biochemical or topographical). Subsequently, the ability of this helically organized medial layer to biochemically respond to a vasoconstrictor was evaluated. These achievements demonstrate the importance of culture parameters on SMC behavior and serve as a critical step toward engineering SRVs that can recapitulate the cytoarchitecture and resulting vasomotor function of natural resistance vessels.

## 2 Results and Discussion

### 2.1 Effects of Biochemical Microenvironment on HASMCs in 2D Culture

**FIGURE 2:**
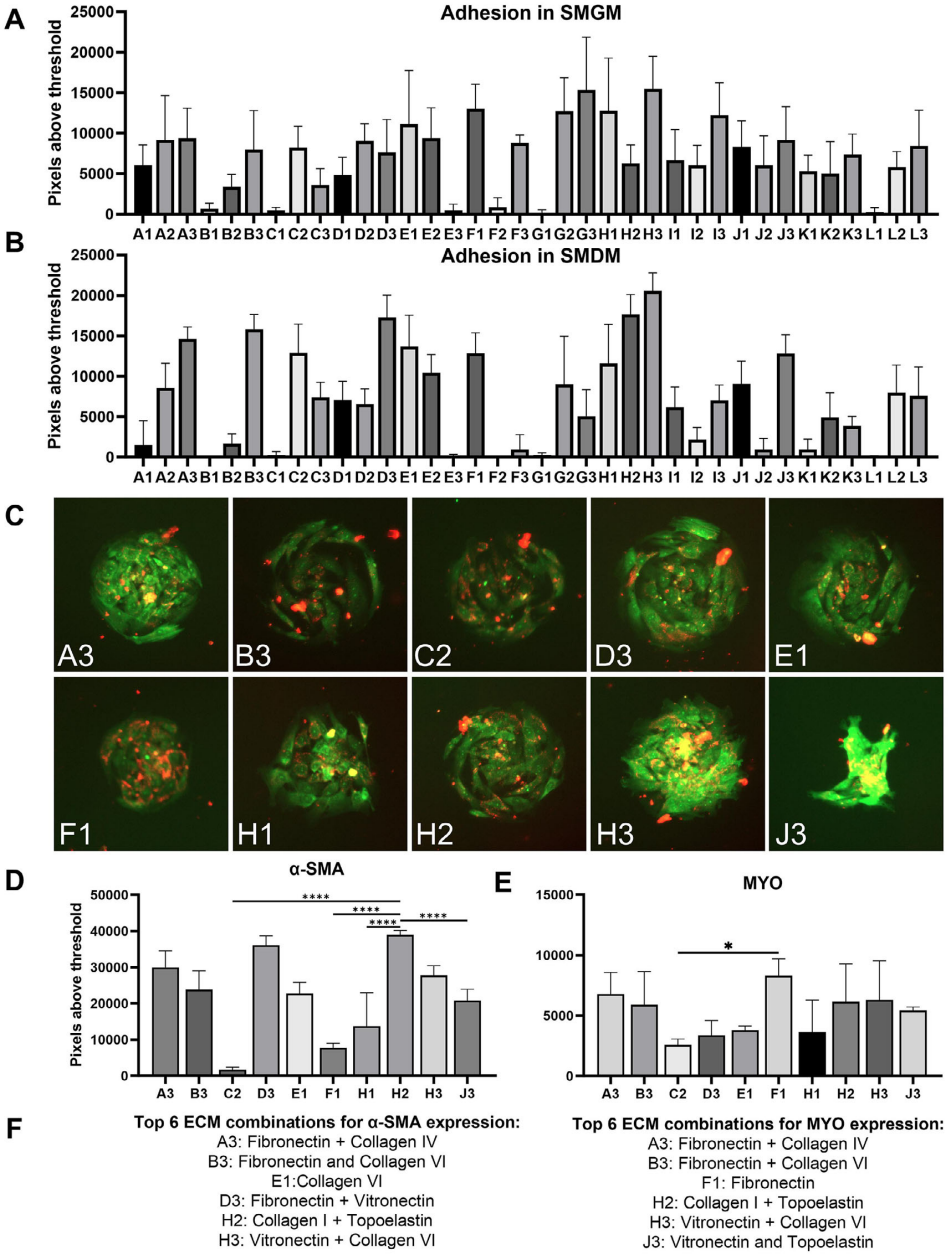
Evaluation of the effects of ECM coating on SMC survival, proliferation, and contractile marker expression. n = 3 for all groups. (A) Quantification of area covered by SMCs after 2 days of culture in SMGM for each ECM condition. (B) Quantification of area covered by SMCs after 2 days of culture in SMGM followed by 4 days in SMDM for each ECM condition. (C) Representative IF staining images for the ten best‐performing ECM conditions after SMGM‐to‐SMDM transition. Green is α‐SMA, red is MYO. Each field of view is 390 µm x 390 µm. (D) Quantification of α‐SMA signal after culture in SMDM. (E) Quantification of MYO signal after culture in SMDM. (F) Identified ECM conditions that induced the most expression of contractile markers. A legend for the composition of each condition can be found in Table S2.

While there is a significant body of evidence to show that ECM proteins are drivers of vascular cell behavior [80, 81, 82], there is conflicting data regarding the specific effects of various ECM proteins. To determine the effects of ECM proteins on attachment and phenotype of human aortic smooth muscle cells (HASMCs), we performed an initial ECM screening assay in which cells were seeded and cultured on surfaces coated with a variety of different ECM proteins and mixtures thereof. ECM surface functionalization was shown to have a profound effect on the behavior of HASMCs (Figure 2 and Table S1). Some ECM conditions resulted in no initial adhesion of cells; others supported adhesion, but the adhered cells were not viable after transitioning from Smooth Muscle Growth Medium (SMGM, which promotes a synthetic phenotype) to Smooth Muscle Differentiation Medium (SMDM, which promotes a contractile phenotype). Other conditions supported cell survival, but the adhered cells either did not appear healthy or did not express α‐smooth muscle actin (α‐SMA) and myosin heavy chain (MYO), as would be expected with the desired contractile phenotype. To quantify the effects of each ECM condition on SMC behavior, an ImageJ macro was used to measure the area covered by cells after initial adhesion in SMGM (Figure 2A), and again after several days of culture in SMDM (Figure 2B). The ten ECM conditions that yielded the most cell coverage following the SMGM‐to‐SMDM transition were selected for further analysis. Immunofluorescence (IF) was then performed to evaluate expression of the contractile markers alpha‐Smooth Muscle Actin (α‐SMA) and Myosin Heavy Chain (MYO) in cells cultured on these ECM combinations (Figure 2C), and the relative expression levels were quantified (Figure 2D,E). Among these ten conditions, four exhibited very low (*p*< 0.0001) α‐SMA expression when compared to the top‐performing condition (H2), and were excluded from further analysis, leaving six candidate conditions. A full report on the statistical analyses for both Figure 2D,E can be found in Tables S4 and S5. The six conditions with the most MYO expression were also identified (Figure 2F). Fibronectin and Vitronectin were the proteins most commonly represented across both groups, and therefore these were selected for evaluation in our 3D culture system.

### 2.2 Introduction of Cells Into Microscale Channels

**FIGURE 3:**
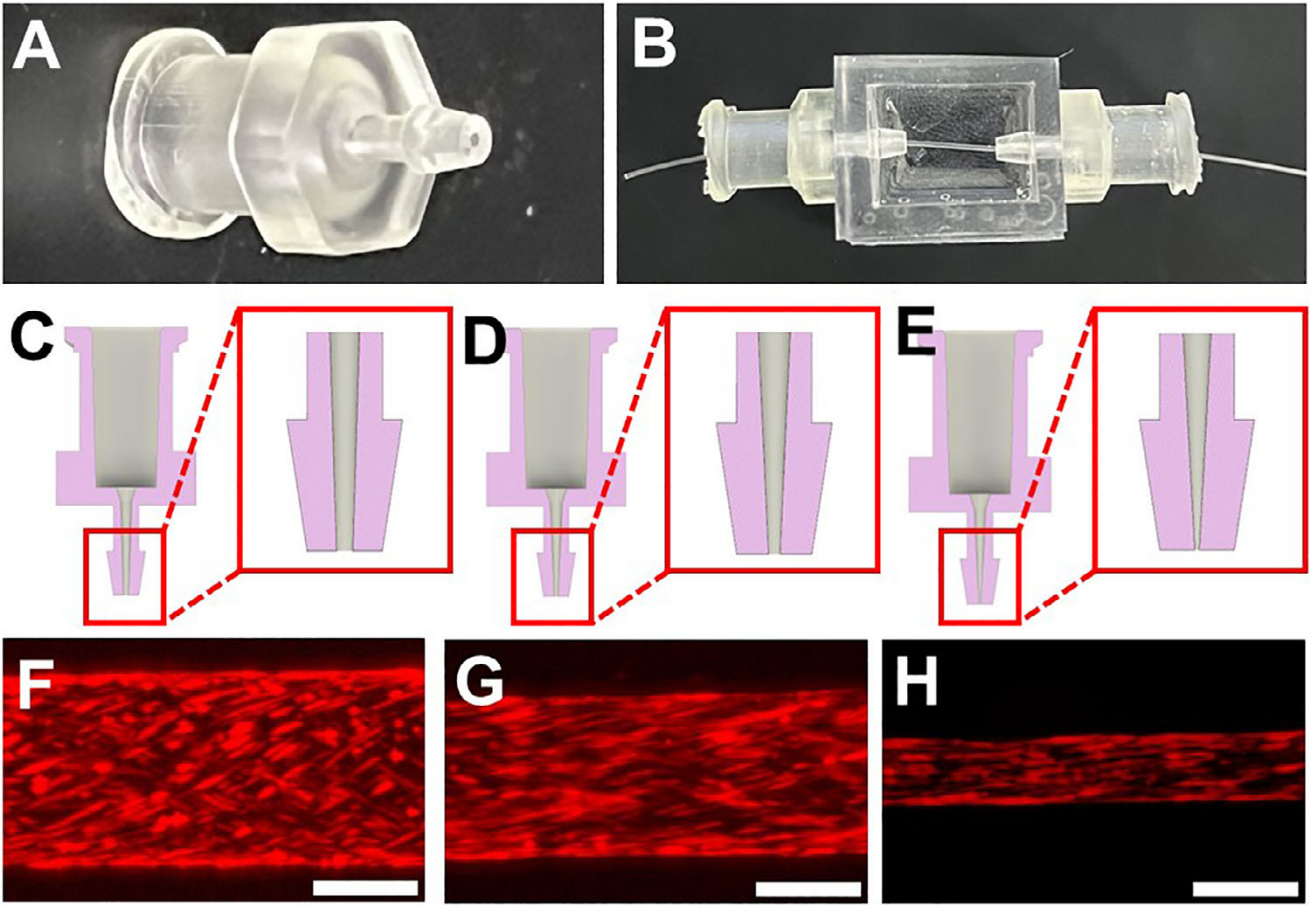
Custom 3D‐printed Luer‐to‐barb connectors (A) in isolation and (B) in assembly ready for channel formation. CAD is shown for barbs with terminal diameters of (C) 400 µm, (D) 330 µm, and (E) 175 µm. SMC‐lined channels formed by suspending templates from these connectors are shown in F, G, and H, respectively. Scale bars are 100 µm.

Next, we seeded HASMCs into straight, microscale channels patterned within a gelatin hydrogel to create a single‐cell‐thick medial layer on the channel wall. The channels were formed by suspending a piece of fishing line (“template”) between 2 Luer connectors, casting gelatin + crosslinker solution over the template, and then removing the template after the gel had set. Initially, we used standard Luer‐to‐barb connectors with a 1/16″ inner diameter (Cole‐Parmer, EW‐50109‐74). However, during cell seeding, this setup consistently caused problems, including highly variable cell seeding density, backflow of cells from the channel into the barb, and extensive accumulation of non‐adhered cells at the barb/channel interface. These accumulated cells and cell debris would be forced into the channels upon media changes or when additional solutions were introduced into the channels. We determined that these issues stemmed primarily from the mismatch between the internal diameter of the barb (∼1.6 mm) and the microchannel (<0.5 mm), which created a discontinuity that disrupted cell flow. To address these issues, we designed and fabricated custom Luer‐to‐barb connectors incorporating internal funnels (Figure 3). The funnel design features a wide base to accommodate firm seating of a standard micropipette tip, and a terminal end internal diameter closely matched to the target channel diameter, with a small amount of clearance to facilitate threading of the monofilament template through the funnel without difficulty.

These funnels enabled consistent delivery of cells into the microchannels at the appropriate density and uniform distribution using only 1–3 µL of cell suspension (just enough to fill the channels), while previously 10s to100s of µL of cell suspension were necessary to get an appreciable number of cells into the channels. Without this excess cell suspension, there was no longer any cell accumulation in the barb. These custom connectors allowed reliable formation of channels across a range of diameters representative of natural SRV diameters, maintained consistent cell seeding density with little backflow, and preserved compatibility with standard Luer‐based fluidic systems. The custom connectors were 3D‐printed using a biocompatible resin and coated with parylene to further ensure biocompatibility. While the goal of this study was to investigate the effects of ECM coating on SMC alignment in a microchannel independent of forces caused by perfusion, to demonstrate that the platform is capable of supporting flow, we perfused with a solution of fluorescent beads at physiologically relevant flow rates using a custom‐built peristaltic pump [83]. The beads were observed flowing through the channels with no leaks or other issues (Figure S1 and Videos S1–S3).

### 2.3 Evaluation of Cell Morphology and Behavior in 3D Culture

**FIGURE 4:**
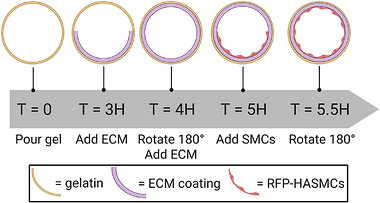
Timeline of cell seeding process.

With a reliable method established for consistent cell seeding into channels, we proceeded with fabricating cell‐lined SRV analogs. We systematically evaluated a range of ECM coatings and cell seeding densities with the aim of optimizing cell adhesion and coverage of the channel surface. Based on the results presented in Section 2.1, four ECM coating conditions were chosen for evaluation: no ECM, Collagen IV alone (C), Collagen IV + Fibronectin (CF), and Collagen IV + Fibronectin + Vitronectin (CFV). All ECM solutions contained equal total protein mass in order to ensure that observed differences in cell behavior were due to the specific proteins included. Seeding densities were varied from 25 000 to 150 000 cells cm^−2^. Channels were formed as described above, coated with the chosen ECM protein solutions, and seeded with HASMCs according to the experimental timeline shown in Figure 4. HASMCs were suspended in SMGM for seeding, and at 6 h post‐seeding, the channels were flushed with SMDM and the devices immersed in SMDM in order to induce a contractile phenotype.

**FIGURE 5:**
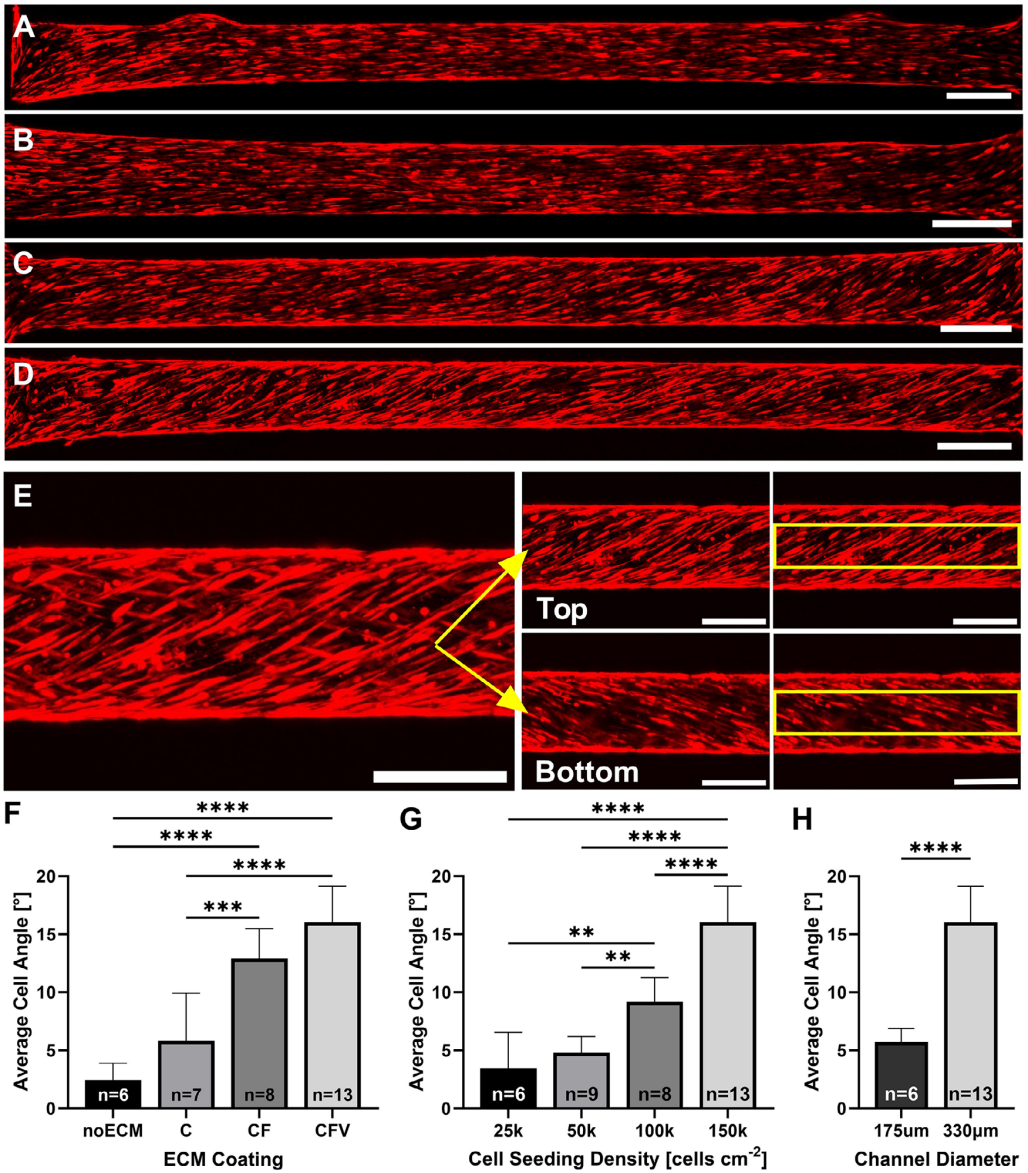
Effect of ECM coating composition on SMC alignment within 330 µm diameter microchannels. Channels were coated with (A) no ECM proteins, (B) Collagen IV, (C) Collagen IV and Fibronectin, or (D) Collagen IV, Fibronectin, and Vitronectin. (E) Preprocessing of images for angle quantification. The full z‐stack is split into upper and lower halves (indicated by yellow arrows), then the central 60% of the channel is cropped (yellow rectangles). The cells within the yellow rectangles are included for angle quantification. (F) Average angle of cells seeded in 330 µm channels at 150 000 cells cm^−2^ varies based on the ECM coating. (G) Average angle of cells seeded in channels coated with CFV varies based on the cell seeding density. (H) Average angle of cells seeded in channels coated with CFV at 150 000 cells cm^−2^ varies based on the channel diameter. All scale bars are 350 µm. Sample number (n) for each experimental condition is labeled on its respective bar.

Following static culture in the channels for five days, HASMCs exhibited significant differences in organization based on the ECM proteins coating the wall of the channel and the initial cell density at seeding (Figure 5). Notably, cells in channels coated with CF or CFV spontaneously aligned around the channel circumference in a helical fashion, while channels coated only with Collagen IV or with no ECM coating resulted in HASMCs aligning longitudinally along the channel axis (Figure 5F). This ECM‐driven organizational effect was further modulated by cell seeding density; low‐density seeding into CFV‐coated channels did not yield significant alignment, while higher density (>100 000 cells cm^−2^) seeding into CFV‐coated channels at higher seeding densities yielded robust alignment (Figure 5G). HASMCs seeded into smaller channels with the same ECM coating and seeding density conditions aligned at a smaller angle (Figure 5H). To ensure that no topographical artifacts were influencing SMC alignment, we performed SEM imaging of the inner surface of the channels and of the filament used to pattern them. Both showed no visible topographical features that could cause the observed SMC alignment (Figure S2). This helical alignment of HASMCs around the channel axis, achieved without the need to specifically pattern binding sites or topographic features, represents a critical advancement toward replicating natural SMC organization within the medial layer of SRVs.

### 2.4 SMC Functionality When Cultured in Microchannels

**FIGURE 6:**
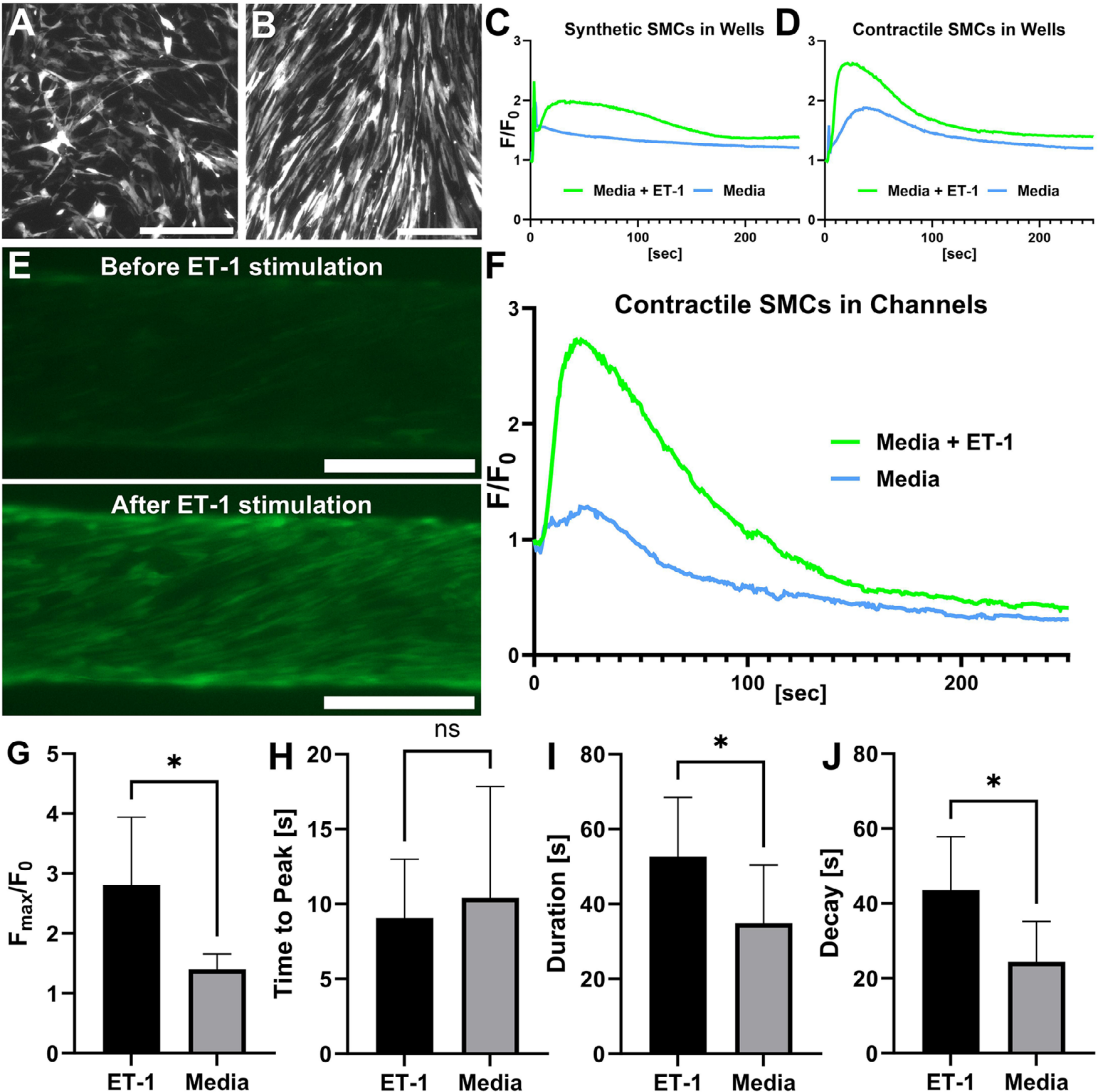
Aligned SMCs in microchannels exhibit an elevated and prolonged Ca^2+^ response when stimulated with vasoconstrictor ET‐1 vs. media refreshment. (A) Synthetic HASMCs and (B) Contractile SMCs in wells exhibit significant morphological differences. (C) Fluorescence response over time for synthetic HASMCs in 6‐well plates treated with either media alone (n = 4) or media spiked with ET‐1 (n = 4). (D) Fluorescence response over time for contractile HASMCs in 6‐well plates treated with either media alone (n = 4) or media spiked with ET‐1 (n = 4). (E) GCaMP6f‐transduced cells in channels exhibit minimal green fluorescence at a baseline (top), and significant fluorescence after stimulation with ET‐1 (bottom), indicating significant influx of Ca^2+^. (F) Fluorescence response over time for cells adhered to the channel wall when treated with either media alone (n = 5) or media spiked with ET‐1 (n = 14). (E) Maximum fluorescence normalized to baseline fluorescence. (F) Time required for fluorescence to peak after stimulation. (G) Duration of the elevated fluorescence response. (H) Time required for fluorescence levels to fall from peak to half‐peak level. All scale bars are 300 µm.

The role of SMCs in regulating the lumen diameter of resistance vessels depends not only on their helical organization around the vessel but also on their responsiveness to vasoactive stimuli. An early and critical step in SMC contraction is an increase in cytosolic Ca^2+^; these ions bind calmodulin, and that complex subsequently activates the contractile machinery in the cells [84]. Therefore, observing Ca^2+^ dynamics is a valuable proxy to evaluate initiation of contractile processes in SMCs without directly observing changes in the diameter of the vessel. To assess such vasoreactive functionality, we evaluated intracellular Ca^2+^ levels in HASMCs upon stimulation with the potent vasoconstrictor endothelin‐1 (ET‐1). For these studies, SMCs were transduced with a lentiviral vector (LV) encoding the calcium indicator GCaMP6f. This modification causes the cells to fluoresce green in response to increases in intracellular Ca^2+^. As expected [60], HASMCs cultured in 6‐well plates in SMGM (i.e., HASMCs cultured in a condition to drive synthetic phenotype) exhibited a compact, rhomboid morphology (Figure 6A), whereas those cultured in SMDM for 5 days (i.e., HASMCs cultured in a condition to drive contractile phenotype) exhibited a spindle‐like morphology (Figure 6B). Modified HASMCs cultured in 6‐well plates in SMGM exhibited minimal response to media refresh and weak response to ET‐1 stimulation (Figure 6C), while HASMCs in 6‐well plates in SMDM for 5 days exhibited strong response curves, with ET‐1 stimulation resulting in a 2.6‐fold increase in peak fluorescence over baseline and media refreshment resulting in a 1.8‐fold increase (Figure 6D). Channels lined with helically aligned modified HASMCs were prepared as previously described, and after 7 days of static culture in SMDM, were perfused with a bolus of either SMDM alone or SMDM supplemented with ET‐1. Similar to the results observed for contractile phenotype HASMCs in a 6‐well plate, media refreshment alone induced a modest 1.4‐fold increase in peak GCaMP6f fluorescence while ET‐1 stimulation resulted in a significantly stronger and more sustained response, with a 2.9‐fold increase in peak GCaMP6f fluorescence, longer time to peak intensity, and prolonged fluorescence duration (Figure 6E–J). SMCs are known to exhibit intracellular Ca^2+^ increases in response to both increased extracellular pH [85] and direct exposure to shear stress [86], both of which would occur with perfusion of fresh media through a microchannel; thus, the modest increase in signal upon media refreshment is unsurprising. Altogether, these data suggest that HASMCs cultured in microchannels in SMDM for 7 days exhibit a robust response to vasoconstrictive signaling and that this response is specific to SMCs in a contractile phenotype.

### 2.5 Formation of an Intimal Layer of Endothelial Cells

**FIGURE 7:**
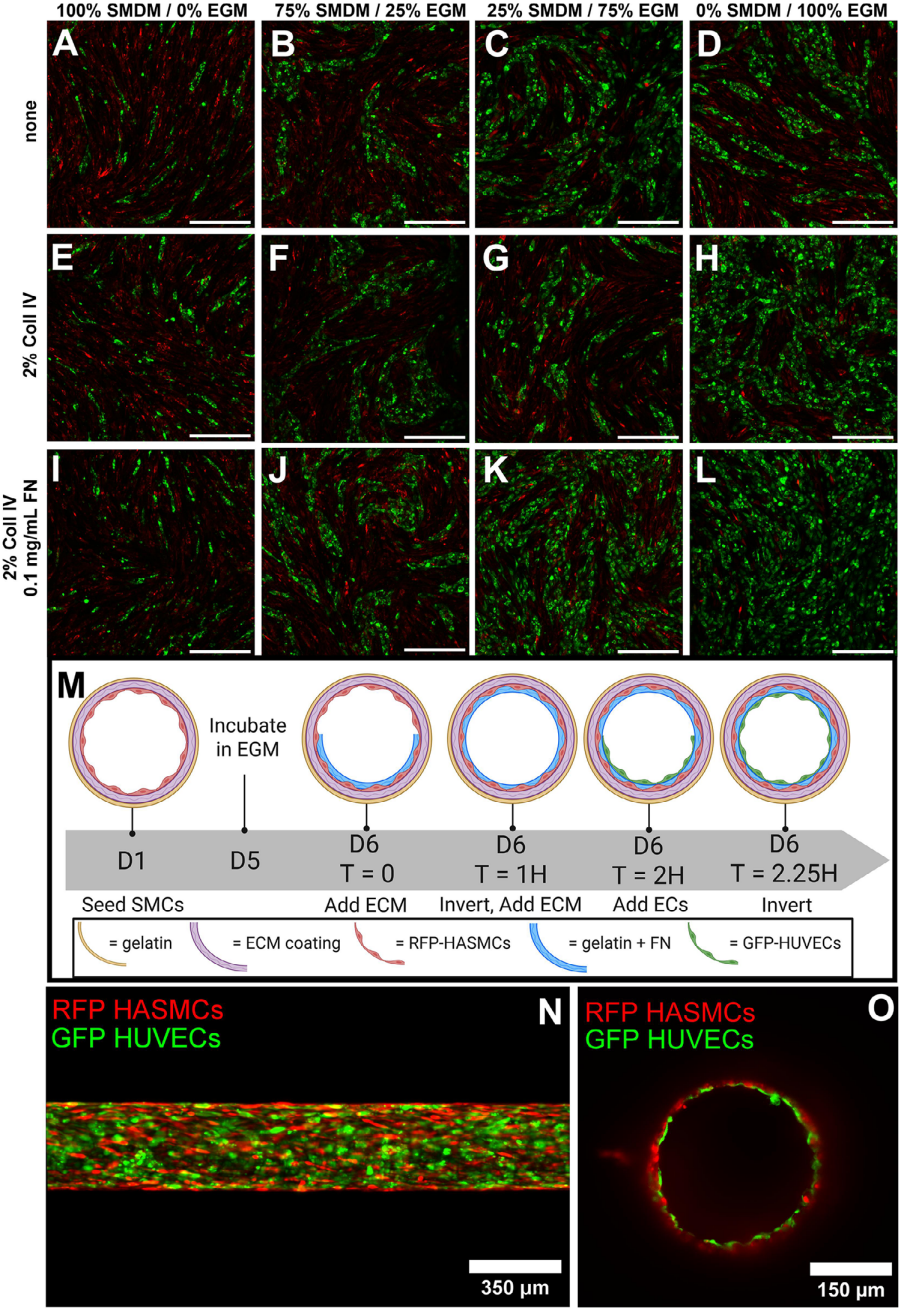
Formation of an intimal layer of ECs in SMC‐lined microchannels. (A‐L) 2D co‐culture of RFP‐HASMCs (red) and GFP‐HUVECs (green). Scale bars are 500 µm. (M) Timeline of the co‐culture seeding process in 3D culture. (N) Co‐cultured HASMCs and HUVECs in a 330 µm channel. (O) Cross‐sectional view showing HUVEC layer intimal to HASMC layer.

An intimal layer was formed by seeding Human Umbilical Vein Endothelial Cells (HUVECs) into the SMC‐lined microchannels. Preliminary 2D co‐culture experiments were conducted to determine the optimal seeding medium for supporting both SMC survival and EC attachment, as well as to assess the effect of adding a layer of proteins between the cell layers. For these 2D experiments, HASMCs were seeded onto a gelatin layer in 24‐well plates and allowed to proliferate in SMGM for 2 days, after which the cells were confluent, and the medium was switched to SMDM for 4 days to promote SMC differentiation. At this point, the culture medium was switched to either pure SMDM, pure endothelial cell growth medium (EGM), or a mixture of the two media types in either a 3:1 or 1:3 ratio. The new medium was allowed to incubate in the wells for 24 h to ensure full permeation of the gelatin slab. Next, a solution of ECM proteins—either gelatin or gelatin + fibronectin (FN)—was added to the wells and allowed to incubate for 1 h. The wells were then washed with their respective media, after which HUVECs were seeded into the wells at 35 000 cells cm^−2^. Both the medium and the presence and composition of proteins between the two cell types were found to influence the extent of HUVEC coverage (Figure 7A–L), but with medium composition appearing to be the more influential factor. For example, within the group with no protein layer (Figure 7A–D), there was increasing EC coverage with increasing proportion of EGM in the seeding medium. This trend was true for all protein layer conditions. The wells in which pure EGM was used to seed HUVECs and gelatin + FN was layered between the cell types saw the greatest extent of HUVEC coverage (Figure 7L), and therefore these conditions were chosen for use in experiments in microchannels. SMC‐lined microchannels were formed as described above, but using barbs with an extended neck (Figure S3) to account for gelatin shrinkage. On D5 post‐SMC seeding, the hydrogel constructs were immersed in EGM for 24 h. A solution of 2% gelatin + 0.1 mg mL^−1^ FN was then introduced into the channel and allowed to incubate for 1 h. The devices were inverted, and then more gelatin + FN solution was added and allowed to incubate for an additional hour. HUVECs were then seeded into the channels at 500 000 cells cm^−1^. After 15 min, the devices were inverted, allowing cells to adhere to both the top and bottom of the channels. A timeline of this process is shown in Figure 7M. These devices were then fixed and imaged using a lightsheet microscope (Figure 7N,O), showing a layer of ECs intimal to the SMC layer.

## 3 Conclusions and Future Directions

The ability of natural resistance vessels to modulate their lumen diameter in response to stimuli (vasoreactivity) is dependent on the presence of a helically aligned layer of contractile SMCs. To properly replicate such vasoreactive function, it is therefore necessary to create a layer of aligned contractile phenotype SMCs in appropriately sized engineered vessels. Most work done thus far toward engineering resistance vessel‐scale microvasculature either produces an SMC layer with inappropriate (not aligned, too thick) architecture or no SMC layer at all. In this study, we have produced a layer of helically aligned SMCs in resistance vessel‐sized channels by optimizing the microenvironment and cell seeding parameters.

We found that HASMCs seeded at high cell seeding density (150 000 cells cm^−1^) into channels coated with appropriately chosen ECM proteins can spontaneously align and exhibit helical organization. The composition of the ECM layer on the channel wall and cell seeding density both had a significant effect on the angle at which the cells align. We also found that this angle is dependent on the channel diameter, with HASMCs seeded in smaller channels aligning at shallower angles. These aligned cells exhibit a robust response to ET‐1–one not seen in SMCs with a synthetic phenotype—suggesting they possess the contractile phenotype necessary for vasoactive function. Achieving this helical orientation of contractile SMCs is a critical first step toward creating vasoreactive resistance vessels (or networks thereof). While optimal hydrogel composition, ECM coating, and seeding density for achieving this orientation may vary for SMCs from different sources, this work provides a framework for determining those optimal conditions. We also found that by layering proteins onto the HASMCs and optimizing the seeding medium, a layer of HUVECs can be added intimal to the helically‐aligned SMC layer.

While the production of a layer of aligned SMCs represents major progress toward engineering vasoactive resistance vessels, there are opportunities to further refine and expand this model. Adding an adventitial layer with embedded fibroblasts would be an important next step, as interplay between cell types is involved in many functions of the microvasculature in vivo. HASMCs in our current system exhibit a significant increase in intracellular Ca^2+^ when stimulated with vasoconstrictor ET‐1, indicating activation of contractile signaling pathways. However, we did not observe measurable changes in channel diameter in response to vasoconstrictor exposure, most likely due to the uniform and relatively high stiffness of the bulk hydrogel surrounding the cells. One next step toward the production of vasoreactive resistance vessels would be to engineer “microtubes” of hydrogel with appropriate distensibility, and then apply the approaches described herein to form a medial layer on such vessels. This would provide the SMCs with a vessel upon which their contractile forces would be expected to cause constriction. Future work could also extend this approach to more complex vascular geometries beyond the straight channels shown here, including branched structures and hierarchical networks that more accurately mimic natural vascular networks. All of the studies presented in this work were performed in static conditions; however, perfusing these vessels with physiological flow conditions (with appropriate pulsatility and in an appropriately distensible vessel) could yield valuable information regarding the effects of cyclic stretch [87, 88] on further enhancement of cell alignment, maturation, and contractile function. Overall, this work represents a critical step forward in creating engineered microvasculature that replicates the structure and function of native resistance vessels. Such vessels can be leveraged to develop more relevant disease models and drug screening platforms, enabling studies that elucidate the role of resistance vessel dysfunction in disease pathogenesis and identify effective therapeutic strategies. Additionally, incorporating resistance vessels with contractile function into implantable engineered tissues would enable these tissues to modulate local vascular resistance and thereby help maintain homeostasis in a manner similar to that of the natural microcirculation.

## 4 Experimental Section

### 4.1 Cells and Reagents

RFP‐expressing HASMCs (Human Aortic Smooth Muscle Cells) were purchased from Angioproteomie (cAP‐0026). GFP‐expressing HUVECs were purchased from Angioproteomie (cAP‐0001GFP). Supplements to make SMGM and SMDM were purchased from ThermoFisher (Smooth Muscle Growth Supplement, S00725 and Smooth Muscle Differentiation Supplement, S0085). Endothelial Cell Growth medium (EGM) was made by spiking DMEM/F12 (Thermofisher, 11320‐033) with 5% fetal bovine serum (FBS) (Thermofisher, A3160402) 10 mM L‐glutamine (Thermofisher, 35050061), 0.75 U mL^−1^ heparin (FisherScientific, BP2425), 50 µg mL^−1^ ascorbic acid (Sigma–Aldrich, A4403‐100MG), 15 ng mL^−1^ IGF‐1 (Thermofisher, 100‐11‐100UG), 5 ng mL^−1^ VEGF (Thermofisher, 100‐20‐100UG), 5 ng mL^−1^ FGFb (Thermofisher, 100–18B‐50UG), 5 ng mL^−1^ EGF (Thermofisher, 100‐15‐100UG), 1 µg mL^−1^ cortisol (Sigma Aldrich, H0888‐1G), and 100 U mL^−1^ pen/strep (Thermofisher, 15140122). To detach HUVECs, 0.25% trypsin‐EDTA (Thermofisher, 25200056) was used, while 0.05% trypsin‐EDTA (Thermofisher, 25300054) was used to detach HASMCs. Collagen Type IV (C7521‐5MG) and Fibronectin (F0895‐1MG) were purchased from Sigma–Aldrich. Vitronectin (5051‐0.1MG) was purchased from Advanced Biomatrix. Gelatin and PBS were purchased from Sigma–Aldrich (G2500‐1KG, P7059‐1L). PDMS elastomer was purchased from Krayden, Inc (DC 184 8.8LB KIT). Antibodies and their dilutions are listed in Table 1. Microbial Transglutaminase (mTg) was purchased from Modernist Pantry (“Moo Gloo [TG]”). A pre‐made lentiviral vector expressing Ca^2+^ indicator GCaMP6f under promoter CMV was purchased from SignaGen Laboratories (LV‐CMV‐GCaMP6f). Prior to experiments, HASMCs were cultured in BioLite flasks or BioLite 6‐well plates, with at least 150 µL of SMGM per cm^2^ of culture dish surface area and media changes every other day; when less media was used – e.g., 8 mL media in a T75 flask‐ HASMCs adopted a semi‐differentiated phenotype, we hypothesize due to partial serum starvation. All experiments were performed with HASMCs between passages 5 and 8 and HUVECs between passages 5 and 10.

**TABLE 1:** Antibody details.

Target	Host	Vendor	Catalog #	Dilution
Smooth Muscle Myosin (MYO)	Mouse	Thermofisher	50‐6400‐80	1:100
Alpha‐Smooth Muscle Actin (SMA)	Mouse	Thermofisher	53‐9760‐80	1:100

### 4.2 Identification of Target ECM Proteins

Various ECM proteins were evaluated for their ability to promote appropriate HASMC adhesion and contractile phenotype on a 2D substrate. Using a commercially available screening array (Advanced BioMatrix, #5170), HASMCs were seeded (50 000 cells mL^−1^ in SMGM) onto a glass slide functionalized with thirty‐six ECM conditions. Cells were imaged with a Zeiss Axio Observer.Z1 wide‐field microscope (at 4, 8, and 12 h post‐seeding) to evaluate the success of initial adhesion to the functionalized surfaces. After cells reached confluence on day 2, the SMGM was replaced with SMDM to promote transition from the synthetic to the contractile phenotype. Imaging was performed regularly in the following days to evaluate the effect of the media transition. After 14 total days of culture (2 in SMGM, 12 in SMDM), the cells were fixed in 4% PFA, and immunofluorescent staining was performed to evaluate the expression of contractile markers alpha‐smooth muscle actin (ɑ‐SMA) and heavy chain smooth muscle myosin (MYO).

An ImageJ macro was used to automatically analyze the images. Each image was opened in ImageJ, and a rolling ball algorithm was used to correct for background illumination. Next, the Triangle method was performed to threshold the image. The number of pixels above the determined threshold value were counted.

### 4.3 Custom Luer‐to‐barb Connector Fabrication

Custom Luer‐to‐barb connectors were designed in Autodesk Fusion and printed using biocompatible photopolymer resin (Liqcreate, Matterhackers) on an SLA 3D printer (Sonic Mini 8K, Phrozen). After printing, the uncured resin was cleaned off with ethanol, and compressed air was used to ensure no residual resin was left in the funnels to prevent clogging. Funnels with terminal diameters ranging from 120–500 µm were successfully fabricated. These connectors were coated with 2 g of parylene‐C using a parylene deposition system (PDS 2010 Labcoter, Specialty Coating Systems) to further ensure biocompatibility. To validate compatibility of the 3D printed Luer‐to‐barb connectors, media was perfused through one of the parylene‐coated connectors for 12 h and then added to 6 well plates with cultured HASMCs. These cells were monitored for one week, with no detrimental effects to cell viability or behavior observed.

### 4.4 Formation of Cell‐Lined Channels Through a Gelatin Hydrogel

Straight microchannels were formed in a hydrogel using nylon monofilament as a removable template. Various line weights of fishing line (KastKing Monofilament, Amazon) were used to achieve channels of various diameters between 200 µm (4 lb line weight) and 500 µm (30 lb line weight). Nylon sewing thread was used to achieve 175 µm channels. The template was strung through two of the funneled Luer‐to‐barb connectors and thereby suspended through a PDMS mold (Figure 3B). Hydrogel precursor solution – 10% w/v gelatin, 2% w/v microbial transglutaminase (mTg) in SMGM—was then poured over the suspended pattern and crosslinked at 37° for 15 min. A solution of ECM proteins—either “C”: 0.55 mg mL^−1^ collagen IV in DI water, “CF”: 0.45 mg mL^−1^ collagen IV + 0.1 mg mL^−1^ fibronectin in DI water, or “CFV”: 0.4 mg mL^−1^ collagen IV, 0.1 mg mL^−1^ fibronectin, and 0.05 mg mL^−1^ vitronectin in DI water—was allowed to incubate inside the channels for 1 h in the upright position, and 1 h in the inverted position. Fresh ECM solution was added when the hydrogel was inverted. After the two 1 h incubation periods, the ECM solution was flushed out of the channels with Hank's Balanced Salt Solution (Gibco, 14175‐095). RFP‐expressing HASMCs suspended in SMGM were then introduced into the channel and allowed to adhere for 6 h, after which the SMGM in the channels was replaced by SMDM via manual flushing with a micropipettor.

### 4.5 Cell Angle Quantification

Five days after seeding, channels were imaged via confocal microscopy using a Zeiss LSM710 microscope with a Fluar 5×/0.25 objective. Multiple overlapping z‐stacks were acquired along the longitudinal axis of the channel in order to capture the entire inner surface. Due to the helical arrangement of the cells around the channel, the cells on the top and bottom of the channel are angled in opposite directions, which made it impossible to measure angle values on a single projection of the full z‐stack. Therefore, each z‐stack was digitally split along the vertical axis to separate the dorsal and ventral halves (“top” and “bottom”), and each half was flattened using a maximum intensity projection. All image analysis was performed in ImageJ. The resulting projections were then tiled longitudinally to generate a continuous composite image of the full inner surface of the channel using Microsoft's Image Composite Editor. We observed that, in these projection images, the cells appeared to be oriented at a shallower angle in the areas near the channel edges. This is likely an artifact of the analysis process; at the periphery of the channel image, the channel surface is orthogonal to the imaging plane, making it impossible to accurately capture the geometry of the cells adhered there given the optical slice thickness. Therefore, only the central 150 µm, in which the channel surface is essentially flat in the imaging plane, was used for analysis. The orientation of individual cells was measured by tracing the cell body manually in ImageJ. Angles were averaged within each dorsal and ventral half‐image, and the two values were then averaged to obtain a single orientation value for each channel.

### 4.6 Calcium Imaging

To assess the functionality of HASMCs in microchannels, we measured the release of intracellular Ca^2+^ in response to stimulation by endothelin‐1 (ET‐1), a potent vasoconstrictor. First, SMCs in a 6‐well plate were modified to express the calcium indicator GCaMP6f through transduction with a lentiviral vector (LV) (SignaGen Laboratories). A single well of a 6‐well plate was incubated with 2 mL of SMGM spiked with LV at a titration of 1.0 × 10^6^ TU mL^−1^ for 12 h, after which the well was washed once with SMGM and then fed with fresh SMGM. After 2 days, the transduced cells were then introduced into channels as described above and left to mature in SMDM for 7 days. Release of intracellular Ca^2+^, as represented by fluorescence intensity, was observed while a bolus of ET‐1 (1.246 µg, 0.5 µmol) in SMDM (0.5 mL) was injected into the channels. All imaging of calcium release was performed using a widefield fluorescence microscope (Zeiss Observer Z.1) with an EC Plan‐Neofluar 5x/0.16 objective, with images taken every 400 ms for a total of 250 s. To characterize the differences in Ca^2+^ response in synthetic vs. contractile SMCs, a similar experiment was conducted with cells grown in 6‐well plates. Cells were seeded into the wells and initially fed with SMGM to promote proliferation. On day 2, LV transduction was performed as described above. On day 3, half of the wells were used to assess the calcium response in HASMCs exhibiting a synthetic phenotype. Release of intracellular Ca^2+^, as represented by fluorescence intensity, was observed as the cells were treated with either a media refresh or with media (2 mL) spiked with ET‐1 (4.984 µg, 2 µmol). For the other half of the wells, the SMGM was replaced with SMDM to promote differentiation into the contractile phenotype. After 5 days in SMDM (day 8), the Ca^2+^ response was evaluated as described above. Four wells were used for each condition.

### 4.7 Co‐Culture of HASMCs and HUVECs

For 2D co‐culture experiments, 24‐well plates were coated with hydrogel precursor solution – 10% w/v gelatin, 2% w/v microbial transglutaminase (mTg) in SMGM—and allowed to crosslink in an incubator at 37°C for 2 h. RFP‐HASMCs were seeded onto the gelatin at 50 000 cells cm^−1^ and allowed to proliferate in SMGM for 2 days. On D3, the media was exchanged for SMDM to promote SMC differentiation. On D6, the media was either refreshed with more SMDM or exchanged for 100% EGM, a mixture of 75% EGM/25% SMDM, or a mixture of 25% EGM/75% SMDM. After 24 h of incubation with the new media, the cells were coated with either a solution of 2% w/v gelatin from porcine skin or 2% w/v gelatin + 0.1 mg mL^−1^ fibronectin, or left with no coating. The protein solutions were made using the respective media formulations already present in the wells. After 1 h of incubation with the protein solutions, each well was washed with its respective media. Next, GFP‐HUVECs were seeded into the wells at 50 000 cells cm^−2^, suspended in the various media formulations already found in each well. 24 h post‐seeding, the media in all wells were switched to 25% EGM/75%SMDM. The wells were imaged with a Zeiss LSM710 microscope with a Fluar 5×/0.25 objective 4 days after HUVEC seeding. The combination of seeding in 100% EGM and coating with gelatin + FN produced the most complete EC coverage (Figure 7L), and so these parameters were used in subsequent experiments in 3D culture. SMC‐lined microchannels were formed as described in Section 4.4, except using the extended‐length 3D‐printed barbs described in Figure S3. The devices were immersed in SMDM beginning at the end of D1. On D5, the devices were immersed in EGM and allowed to incubate for 24 h to ensure complete replacement of the SMDM. A solution of 2% w/v gelatin + 0.1 mg mL^−1^ fibronectin in EGM was introduced into each channel and allowed to incubate for 1 h. The devices were flipped, a new gelatin + FN solution was added, and allowed to incubate for another hour. The channels were flushed with HBSS. GFP‐HUVECs suspended in EGM were then introduced into the channels at 500 000 cells cm^2^ and allowed to adhere for 15 min, after which the devices were inverted. After 4 h, the channels were flushed with EGM to remove non‐adhered cells. The devices were fixed with 4% PFA 24 h after EC seeding and imaged using a Zeiss Z.1 Lightsheet microscope.

### 4.8 Perfusion of Microchannels

HASMC‐lined channels were fixed by perfusing the channel with a bolus of 4% PFA (Thermofisher, J61899.AP), followed by immersion of the entire device in PFA for 1 h. The device was attached to 1/16″ ID Nalgene silicone tubing (Thermofisher, 8600‐0020) using a standard male luer‐to‐barb fitting (Cole‐Parmer, EW‐50110‐11). The tubing was attached to our custom‐built peristaltic pump, and a reservoir containing PBS spiked at a 1:100 ratio with 2 µm‐diameter yellow‐green FluoSpheres (Thermofisher, F8827). The spiked PBS was perfused through the channel beginning at 1 µL min^−1^ while the device was imaged using a Zeiss LSM710 confocal microscope with a Fluar 5×/0.25 objective. The device was continuously observed for any signs of leaks or other issues. The flow rate was gradually increased to 500 µL min^−1^ with no leaks observed.

### 4.9 SEM Imaging

To prepare constructs for imaging via Scanning Electron Microscopy (SEM), microchannels were formed as described in Section 4.4, up to and including coating with “CFV” ECM coating, but without introducing any cells. A 34 g stainless steel wire was then inserted into the channel and used to slice the gelatin block in half, exposing the inner surface of the channel. Samples were dehydrated through a graded ethanol series (30%, 50%, 70%, 80%, 85%, 90%, 95%,100%, 100%, 100%, 100%), incubating each step for 20 min, with the final change held for 12 h. After dehydration, the samples were critical point dried using a Tousimis PVT‐3D critical point drier. After removing the bulk ethanol, the samples were allowed to equilibrate in liquid CO2 for an additional hour, and the fluid was fully exchanged before heating the sample to the critical point. After critical point drying, the hydrogel samples were mounted on carbon tabs and sputter coated with platinum. Additionally, a strand of the monofilament used for patterning was cut approximately 10 mm in length and placed on a carbon tab, followed by sputter coating with platinum. Samples were imaged in a Zeiss Crossbeam 550 FIB‐SEM at 5 keV using the SEM column.

### 4.10 Statistical Analysis

All Statistical analyses were performed using Prism (GraphPad, Version 10.0.0). All experiments were conducted at least two times. For comparisons between 2 groups, an unpaired *t*‐test was performed to analyze data. For comparisons between 3 or more groups, a one‐way ANOVA test with Tukey test was performed. For all comparisons, statistical significance is indicated by *(*p*≤ 0.05), **(*p*≤ 0.01), ***(*p*≤ 0.001), or ****(*p*≤ 0.0001).

## Conflicts of Interest

The authors declare no conflicts of interest.

## Supporting information




**Supporting File 1**: adhm71332‐sup‐0001‐SuppMat.docx.

**Supporting File 2**: adhm71332‐sup‐0002‐VideoS1‐S3.zip.

## Data Availability

The data that support the findings of this study are openly available in Zenodo at http://doi.org/10.5281/zenodo.17832843, reference number 17832843.
