# Supplementary material for: Spontaneous Helical Alignment of Smooth Muscle Cells to Form a Medial Layer for Engineered Microvasculature
Source: Adv Healthc Mater. 2026 Jun 29;15(27):e05938. doi: 10.1002/adhm.202505938 (PMC13378480; doi:10.1002/adhm.202505938)
Supplement: Supplementary file 1 — Supporting File 1: adhm71332‐sup‐0001‐SuppMat.docx. [file ADHM-15-0-s001.docx]

Supplementary Information: Spontaneous Helical Alignment of Smooth Muscle Cells within the Medial Layer of Engineered Microvasculature

Victoria D. Vest, Katherine J. Young, Isabella K. Holtz, Mark Mc Veigh, James D. West and Leon M. Bellan

**Table S1.** Qualitative evaluation of the effects of various ECM coatings on ECM behavior.

*
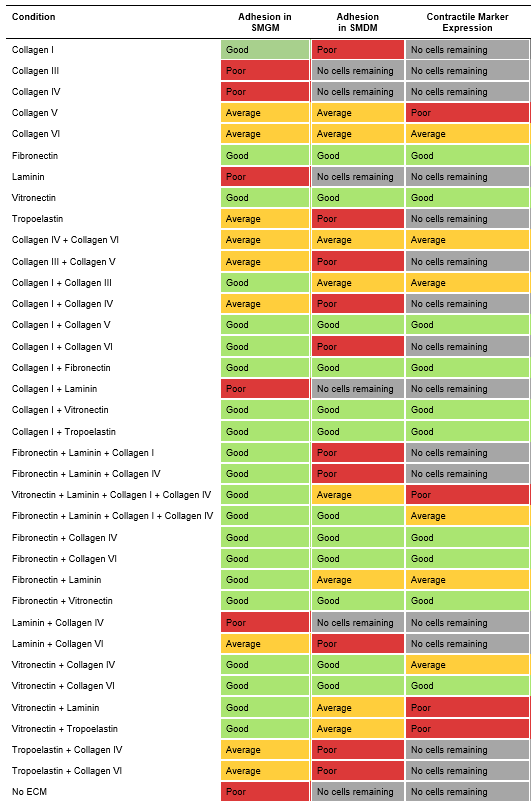
*

**Table S2.** ECM coatings for each condition in Figure 2 (according to the vendor datasheet)


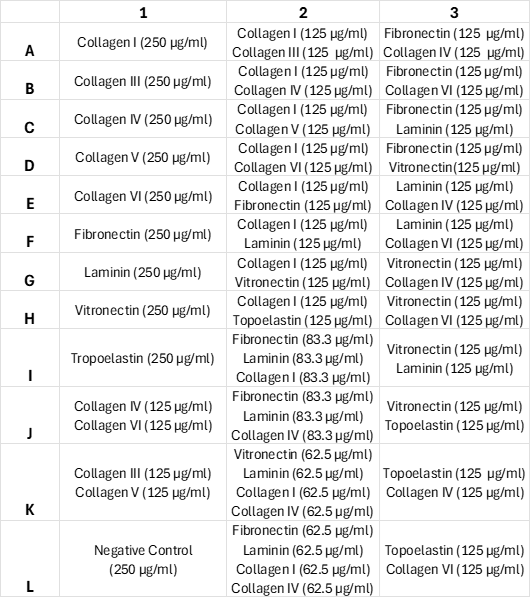


**Table S3.**  Representative images of SMCs from ECM screening array. Field of view for each image is 390 µm x 390 µm
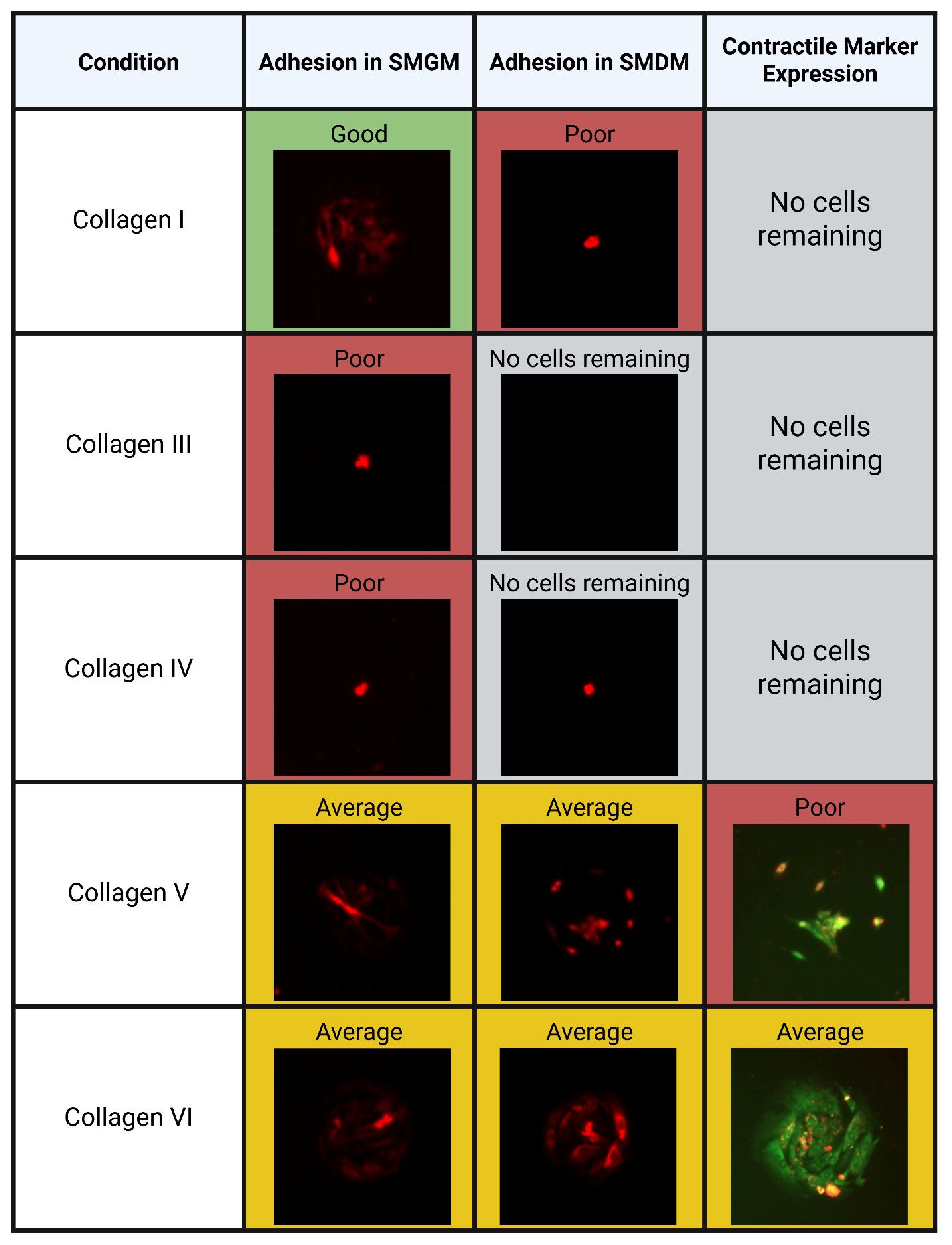


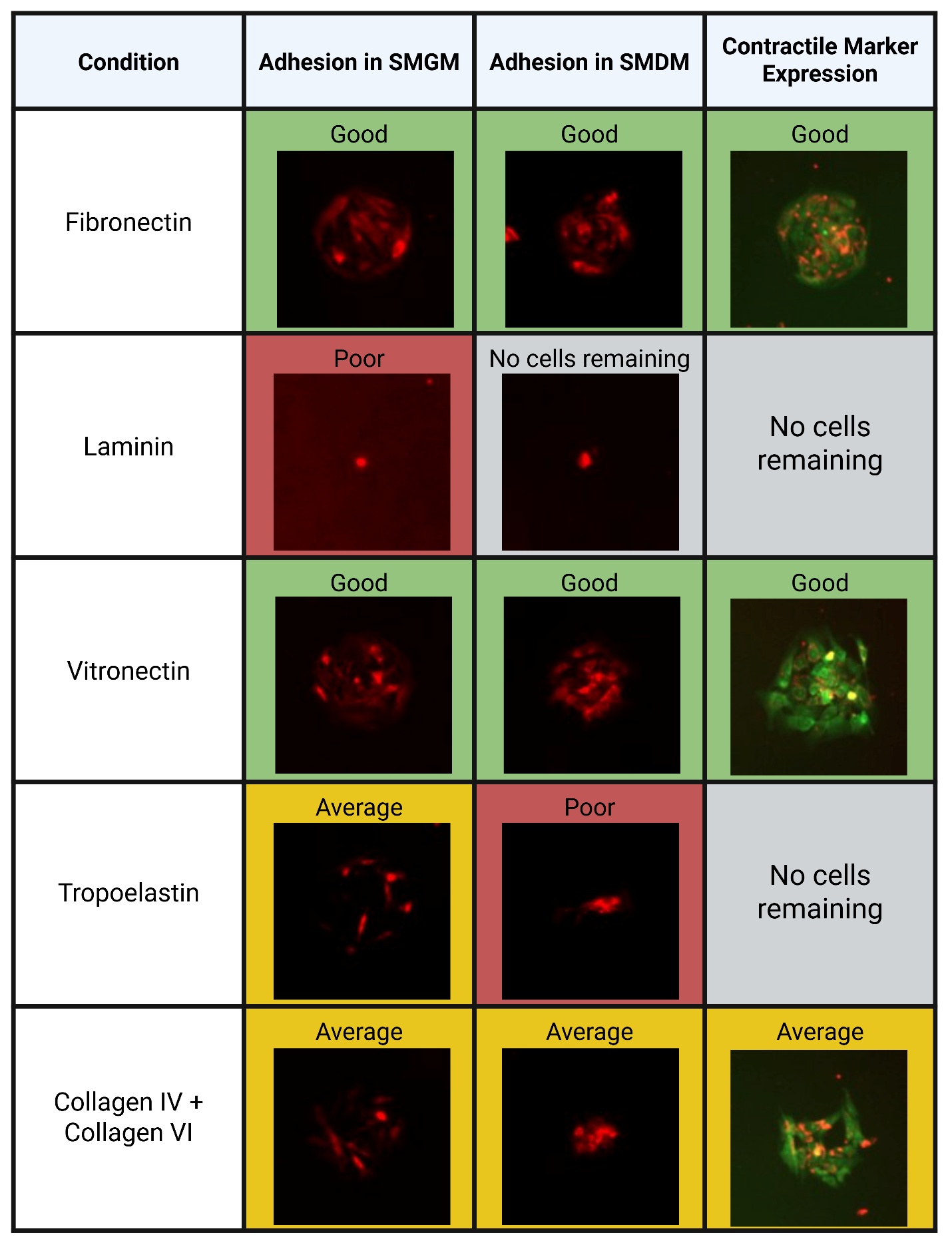

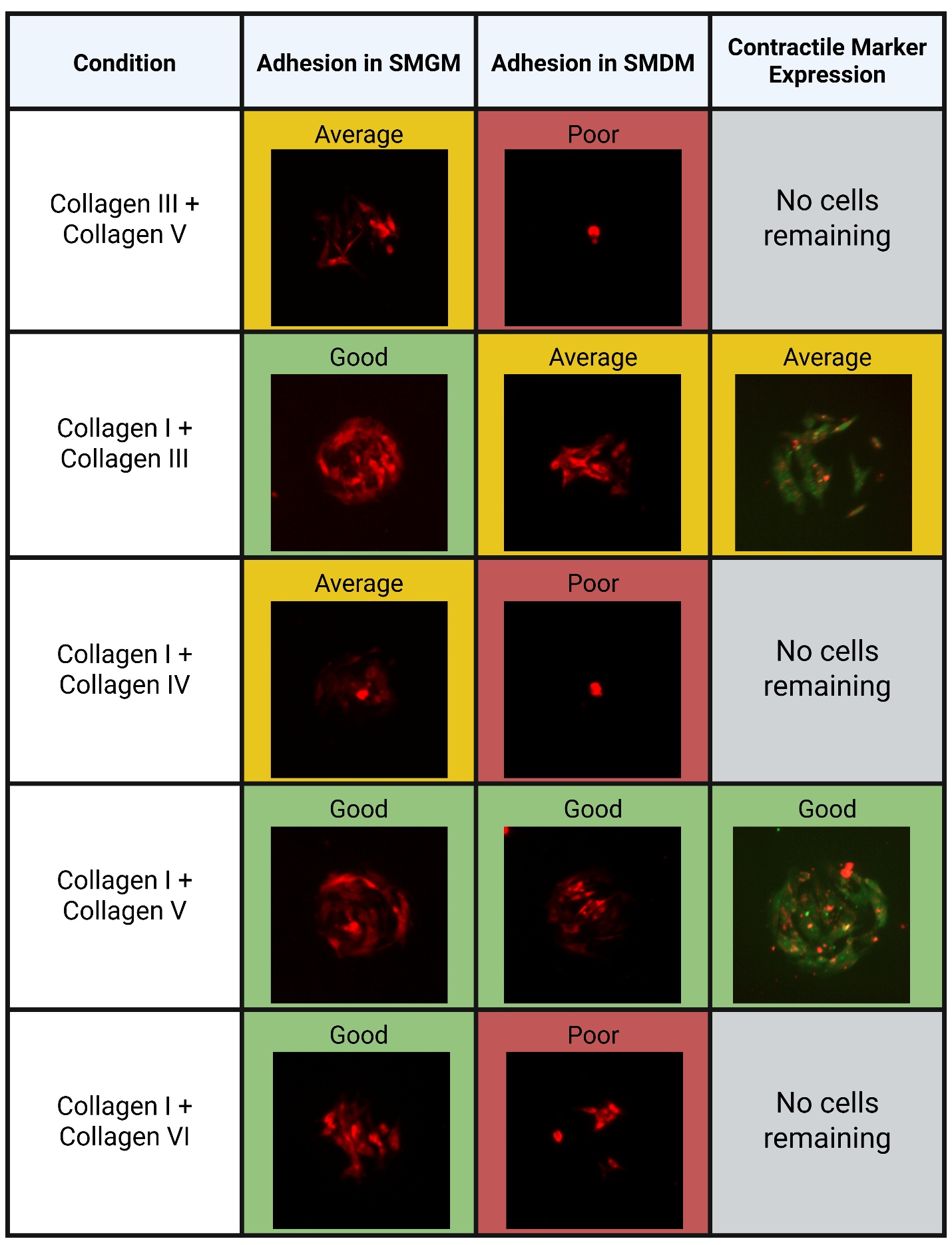

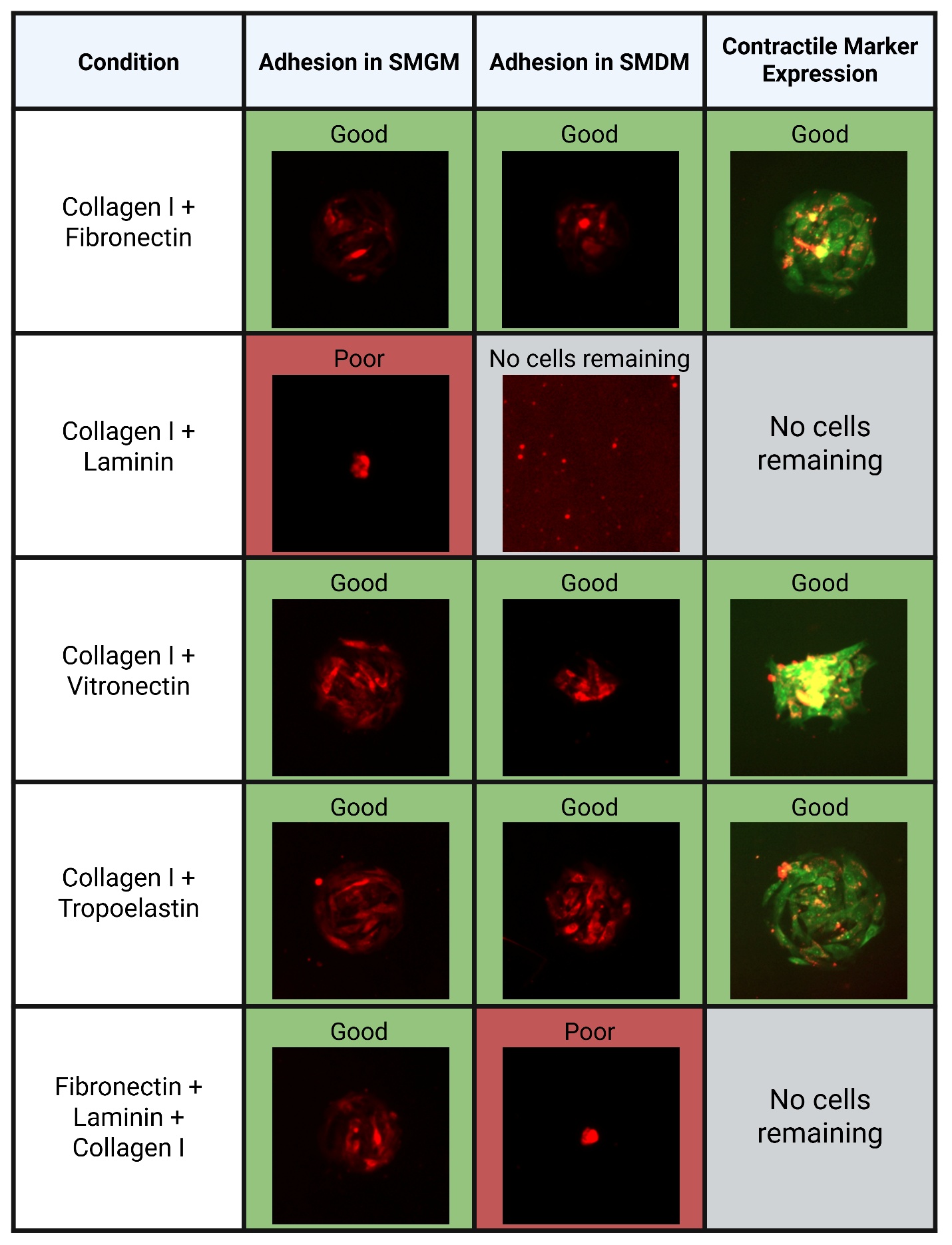

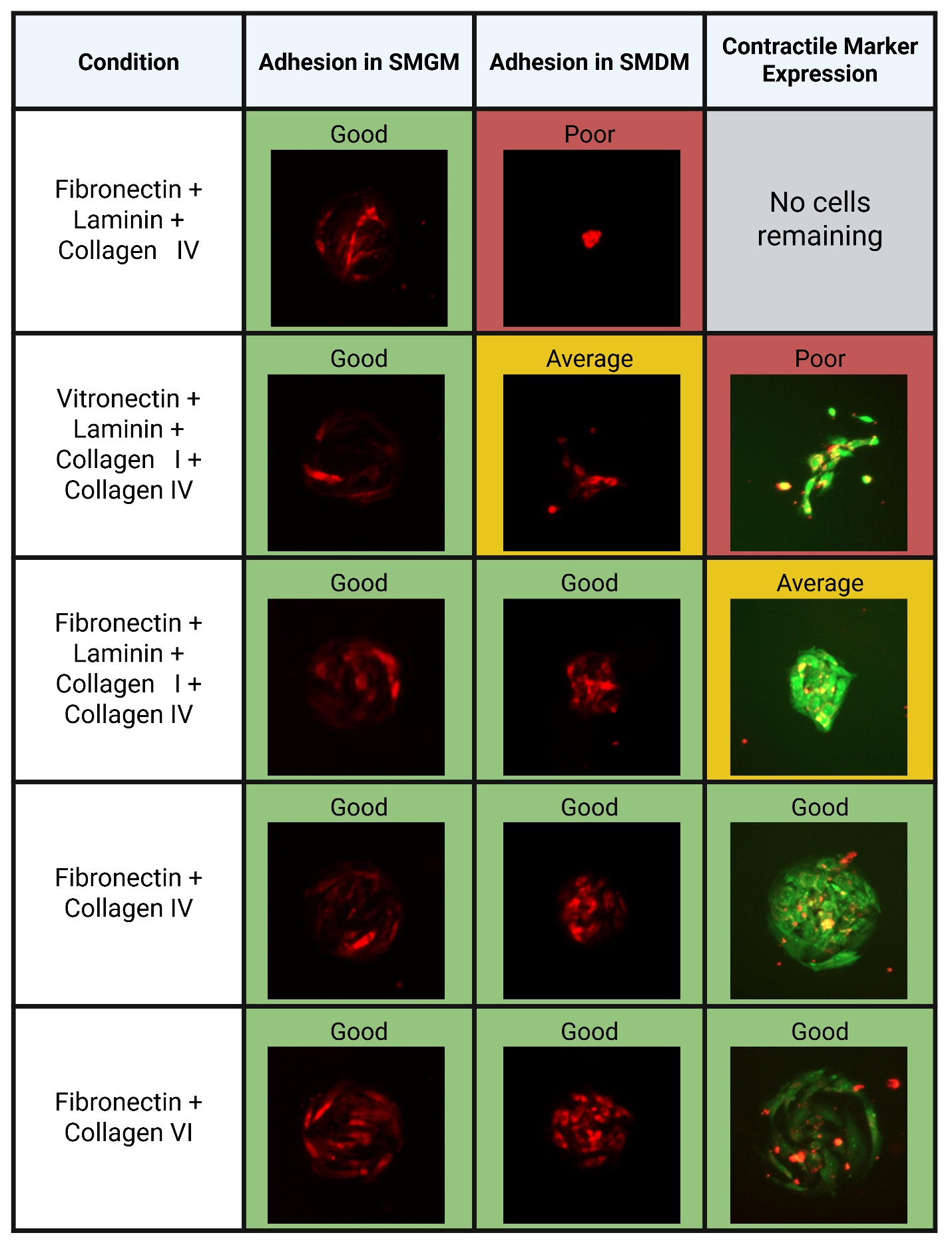

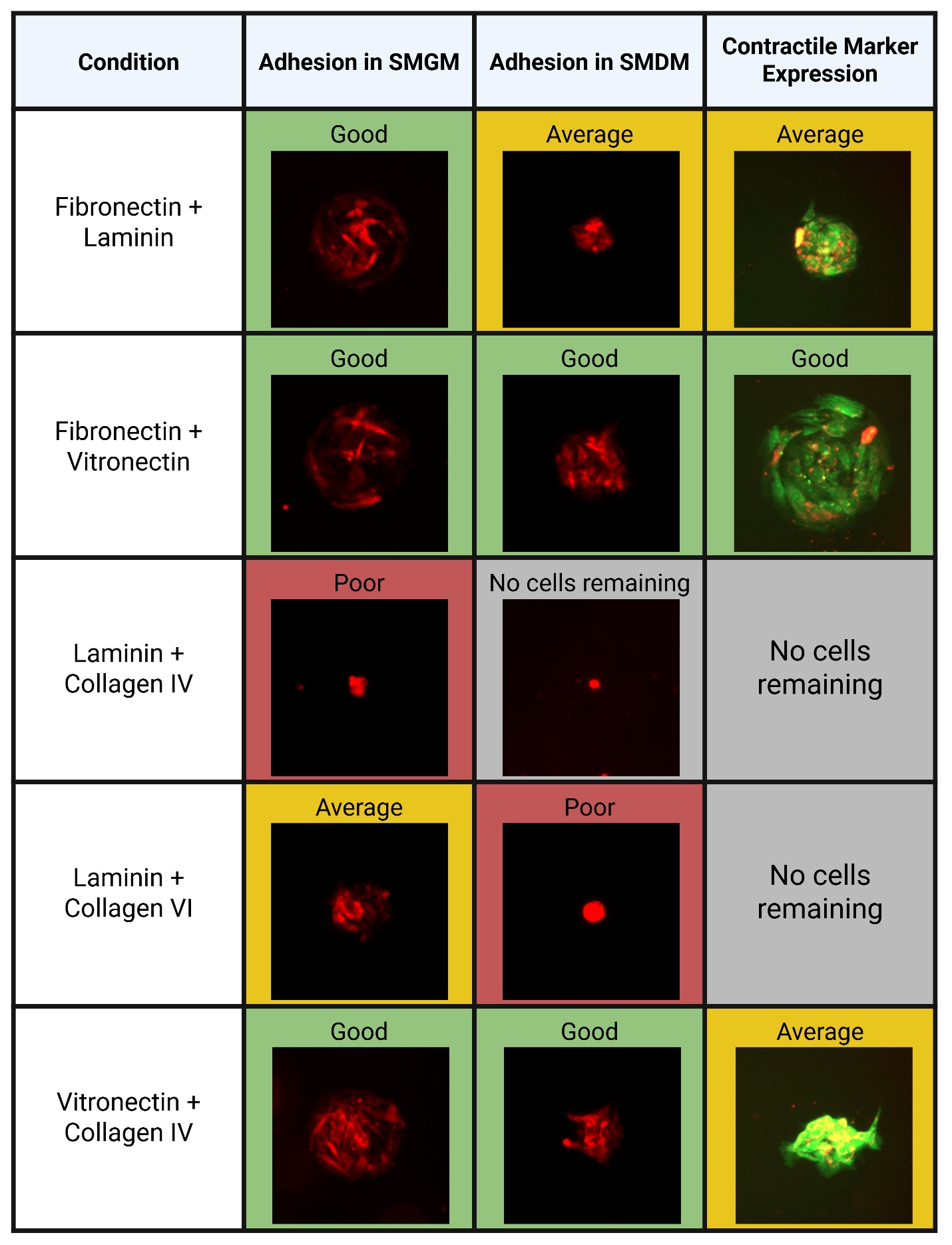

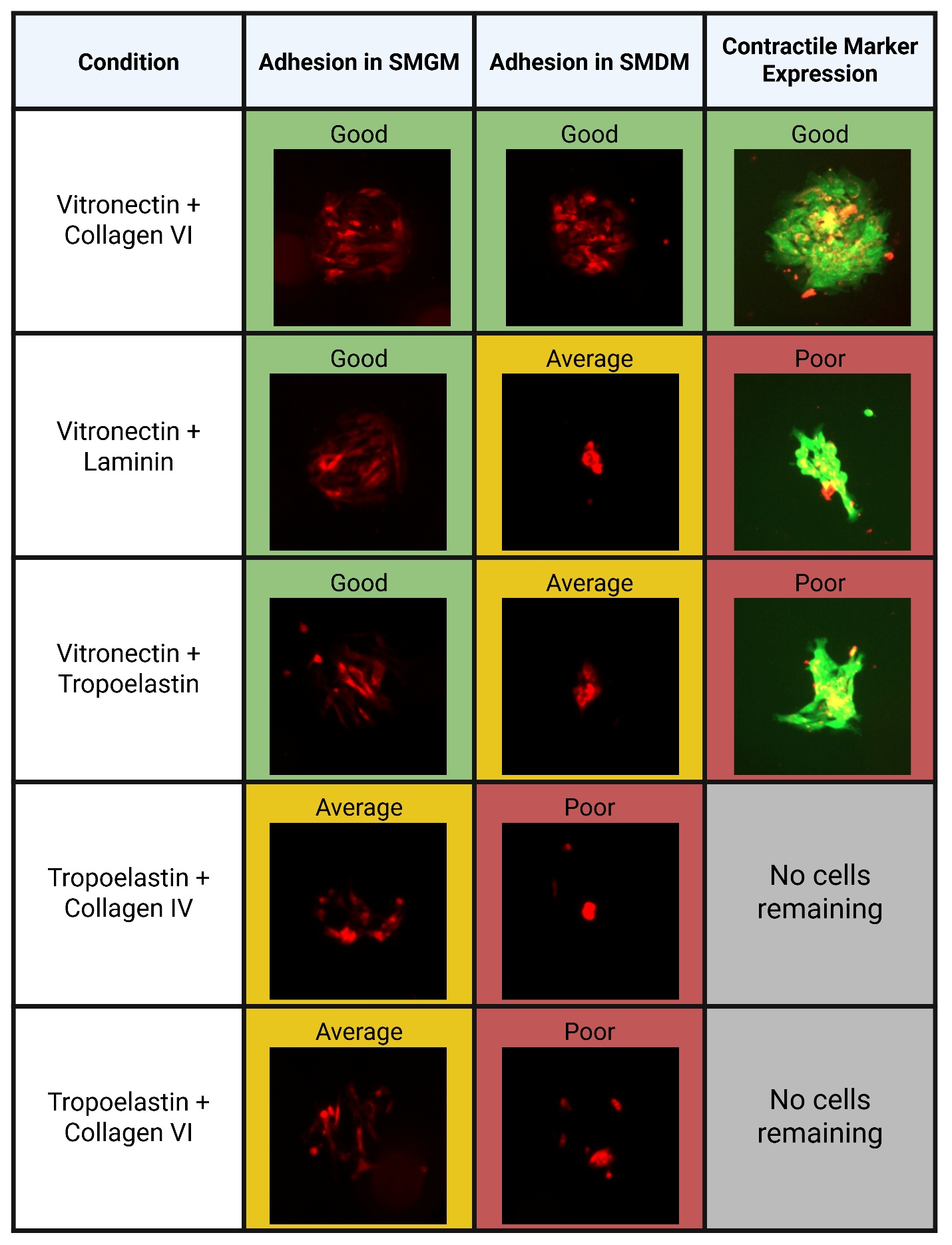


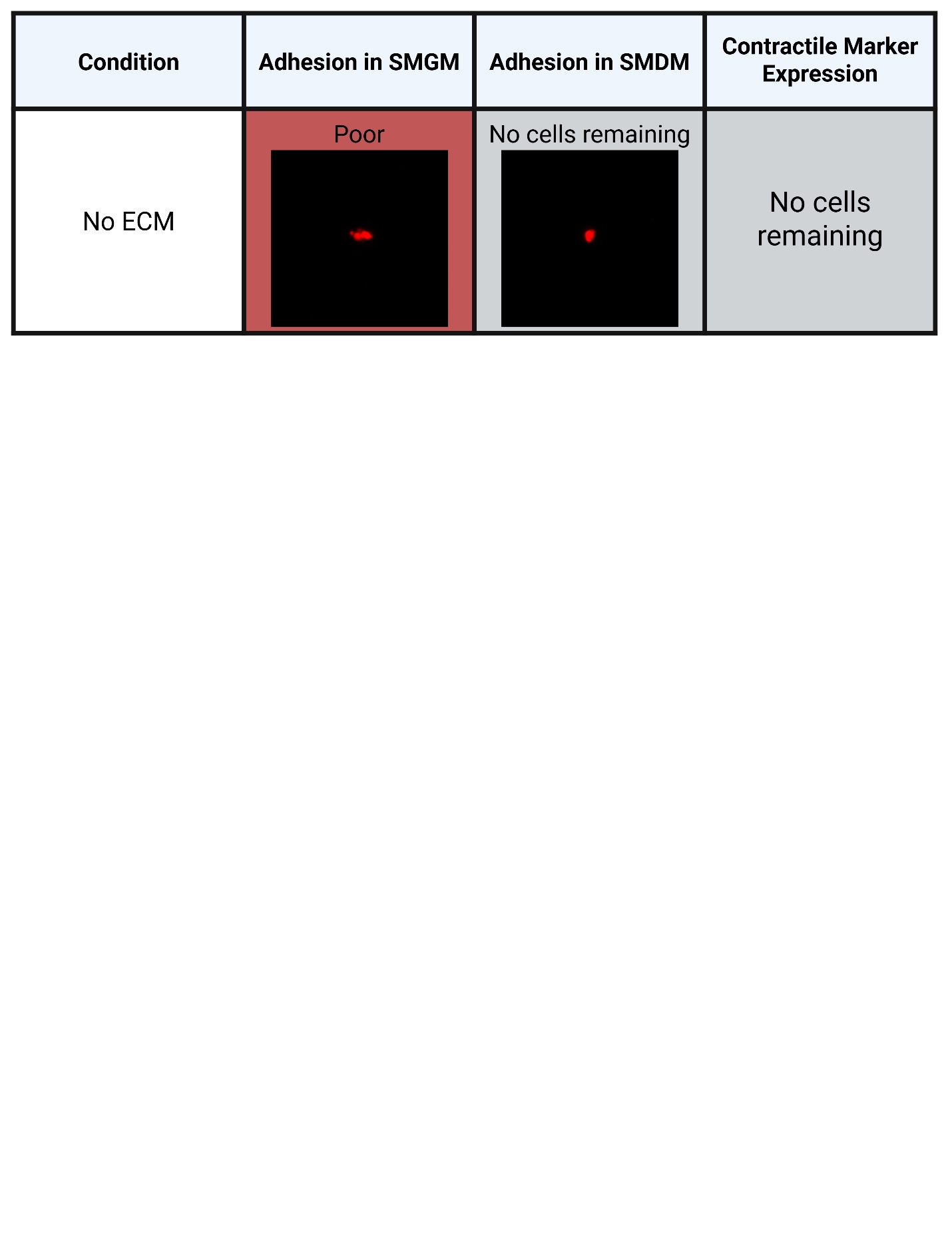


**Table S4**. Statistical data for Myosin signal

| Tukey's multiple comparisons test | Mean Diff. | 95.00% CI of diff. | Summary | Adjusted P Value |
| --- | --- | --- | --- | --- |
| A3 vs. B3 | 873.3 | -4076 to 5822 | ns | 0.9998 |
| A3 vs. C2 | 4207 | -742.4 to 9156 | ns | 0.1499 |
| A3 vs. D3 | 3392 | -1557 to 8341 | ns | 0.3954 |
| A3 vs. E1 | 2976 | -1973 to 7925 | ns | 0.5725 |
| A3 vs. F1 | -1535 | -6484 to 3415 | ns | 0.9853 |
| A3 vs. H1 | 3129 | -1820 to 8078 | ns | 0.5053 |
| A3 vs. H2 | 633.5 | -4316 to 5583 | ns | >0.9999 |
| A3 vs. H3 | 465.0 | -4484 to 5414 | ns | >0.9999 |
| A3 vs. J3 | 1341 | -3608 to 6290 | ns | 0.9943 |
| B3 vs. C2 | 3334 | -1616 to 8283 | ns | 0.4190 |
| B3 vs. D3 | 2519 | -2430 to 7468 | ns | 0.7669 |
| B3 vs. E1 | 2103 | -2846 to 7052 | ns | 0.9009 |
| B3 vs. F1 | -2408 | -7357 to 2541 | ns | 0.8083 |
| B3 vs. H1 | 2256 | -2693 to 7205 | ns | 0.8586 |
| B3 vs. H2 | -239.8 | -5189 to 4709 | ns | >0.9999 |
| B3 vs. H3 | -408.3 | -5357 to 4541 | ns | >0.9999 |
| B3 vs. J3 | 467.8 | -4481 to 5417 | ns | >0.9999 |
| C2 vs. D3 | -814.5 | -5764 to 4135 | ns | 0.9999 |
| C2 vs. E1 | -1231 | -6180 to 3719 | ns | 0.9970 |
| C2 vs. F1 | -5741 | -10690 to -792.1 | * | 0.0133 |
| C2 vs. H1 | -1078 | -6027 to 3871 | ns | 0.9989 |
| C2 vs. H2 | -3573 | -8522 to 1376 | ns | 0.3271 |
| C2 vs. H3 | -3742 | -8691 to 1207 | ns | 0.2704 |
| C2 vs. J3 | -2866 | -7815 to 2083 | ns | 0.6214 |
| D3 vs. E1 | -416.0 | -5365 to 4533 | ns | >0.9999 |
| D3 vs. F1 | -4927 | -9876 to 22.35 | ns | 0.0518 |
| D3 vs. H1 | -263.3 | -5212 to 4686 | ns | >0.9999 |
| D3 vs. H2 | -2759 | -7708 to 2190 | ns | 0.6681 |
| D3 vs. H3 | -2927 | -7876 to 2022 | ns | 0.5942 |
| D3 vs. J3 | -2051 | -7000 to 2898 | ns | 0.9133 |
| E1 vs. F1 | -4511 | -9460 to 438.4 | ns | 0.0976 |
| E1 vs. H1 | 152.8 | -4796 to 5102 | ns | >0.9999 |
| E1 vs. H2 | -2343 | -7292 to 2606 | ns | 0.8308 |
| E1 vs. H3 | -2511 | -7460 to 2438 | ns | 0.7699 |
| E1 vs. J3 | -1635 | -6584 to 3314 | ns | 0.9776 |
| F1 vs. H1 | 4664 | -285.6 to 9613 | ns | 0.0778 |
| F1 vs. H2 | 2168 | -2781 to 7117 | ns | 0.8840 |
| F1 vs. H3 | 2000 | -2950 to 6949 | ns | 0.9246 |
| F1 vs. J3 | 2876 | -2074 to 7825 | ns | 0.6171 |
| H1 vs. H2 | -2496 | -7445 to 2454 | ns | 0.7760 |
| H1 vs. H3 | -2664 | -7613 to 2285 | ns | 0.7084 |
| H1 vs. J3 | -1788 | -6737 to 3161 | ns | 0.9607 |
| H2 vs. H3 | -168.5 | -5118 to 4781 | ns | >0.9999 |
| H2 vs. J3 | 707.5 | -4242 to 5657 | ns | >0.9999 |
| H3 vs. J3 | 876.0 | -4073 to 5825 | ns | 0.9998 |

**Table S5**. Statistical data for α-SMA signal

| Tukey's multiple comparisons test | Mean Diff. | 95.00% CI of diff. | Summary | Adjusted P Value |
| --- | --- | --- | --- | --- |
| A3 vs. B3 | 6107 | -3856 to 16069 | ns | 0.5472 |
| A3 vs. C2 | 28338 | 18375 to 38301 | **** | <0.0001 |
| A3 vs. D3 | -6152 | -16115 to 3811 | ns | 0.5372 |
| A3 vs. E1 | 7197 | -2766 to 17159 | ns | 0.3266 |
| A3 vs. F1 | 22171 | 12208 to 32134 | **** | <0.0001 |
| A3 vs. H1 | 16248 | 6285 to 26211 | *** | 0.0002 |
| A3 vs. H2 | -9002 | -18965 to 960.7 | ns | 0.1033 |
| A3 vs. H3 | 2200 | -7763 to 12162 | ns | 0.9988 |
| A3 vs. J3 | 9176 | -787.0 to 19139 | ns | 0.0910 |
| B3 vs. C2 | 22232 | 12269 to 32195 | **** | <0.0001 |
| B3 vs. D3 | -12259 | -22222 to -2296 | ** | 0.0072 |
| B3 vs. E1 | 1090 | -8873 to 11053 | ns | >0.9999 |
| B3 vs. F1 | 16064 | 6101 to 26027 | *** | 0.0002 |
| B3 vs. H1 | 10141 | 178.3 to 20104 | * | 0.0434 |
| B3 vs. H2 | -15109 | -25072 to -5146 | *** | 0.0005 |
| B3 vs. H3 | -3907 | -13870 to 6056 | ns | 0.9362 |
| B3 vs. J3 | 3070 | -6893 to 13032 | ns | 0.9860 |
| C2 vs. D3 | -34491 | -44453 to -24528 | **** | <0.0001 |
| C2 vs. E1 | -21142 | -31105 to -11179 | **** | <0.0001 |
| C2 vs. F1 | -6168 | -16130 to 3795 | ns | 0.5339 |
| C2 vs. H1 | -12091 | -22053 to -2128 | ** | 0.0084 |
| C2 vs. H2 | -37341 | -47303 to -27378 | **** | <0.0001 |
| C2 vs. H3 | -26139 | -36102 to -16176 | **** | <0.0001 |
| C2 vs. J3 | -19162 | -29125 to -9199 | **** | <0.0001 |
| D3 vs. E1 | 13349 | 3386 to 23312 | ** | 0.0027 |
| D3 vs. F1 | 28323 | 18360 to 38286 | **** | <0.0001 |
| D3 vs. H1 | 22400 | 12437 to 32363 | **** | <0.0001 |
| D3 vs. H2 | -2850 | -12813 to 7113 | ns | 0.9916 |
| D3 vs. H3 | 8352 | -1611 to 18315 | ns | 0.1621 |
| D3 vs. J3 | 15328 | 5365 to 25291 | *** | 0.0004 |
| E1 vs. F1 | 14974 | 5011 to 24937 | *** | 0.0006 |
| E1 vs. H1 | 9051 | -911.7 to 19014 | ns | 0.0997 |
| E1 vs. H2 | -16199 | -26162 to -6236 | *** | 0.0002 |
| E1 vs. H3 | -4997 | -14960 to 4966 | ns | 0.7810 |
| E1 vs. J3 | 1980 | -7983 to 11942 | ns | 0.9995 |
| F1 vs. H1 | -5923 | -15886 to 4040 | ns | 0.5875 |
| F1 vs. H2 | -31173 | -41136 to -21210 | **** | <0.0001 |
| F1 vs. H3 | -19971 | -29934 to -10008 | **** | <0.0001 |
| F1 vs. J3 | -12995 | -22958 to -3032 | ** | 0.0037 |
| H1 vs. H2 | -25250 | -35213 to -15287 | **** | <0.0001 |
| H1 vs. H3 | -14048 | -24011 to -4085 | ** | 0.0014 |
| H1 vs. J3 | -7072 | -17035 to 2891 | ns | 0.3491 |
| H2 vs. H3 | 11202 | 1239 to 21165 | * | 0.0181 |
| H2 vs. J3 | 18178 | 8215 to 28141 | **** | <0.0001 |
| H3 vs. J3 | 6977 | -2986 to 16939 | ns | 0.3669 |

Figure S1 demonstrates the feasibility of perfusing the microchannel devices. HASMC-lined channels were fixed by perfusing the channel with a bolus of 4% PFA (Thermofisher, J61899.AP), followed by immersion of the entire device in PFA for 1 hour. The device was attached to 1/16” ID Nalgene silicone tubing (Thermofisher, 8600-0020) using a standard male luer-to-barb fitting (Cole-Parmer, EW-50110-11). The tubing was attached to our custom-built peristaltic pump and a reservoir containing PBS spiked at a 1:100 ratio with 2 µm-diameter yellow-green FluoSpheres (Thermofisher, F8827). The spiked PBS was perfused through the channel beginning at 1 µL min^-1^ while the device was imaged using a Zeiss LSM710 confocal microscope with a Fluar 5x/0.25 objective. The device was continuously observed for any signs of leaks or other issues. The flow rate was gradually increased to 500 µL min^-1^ with no leaks observed.


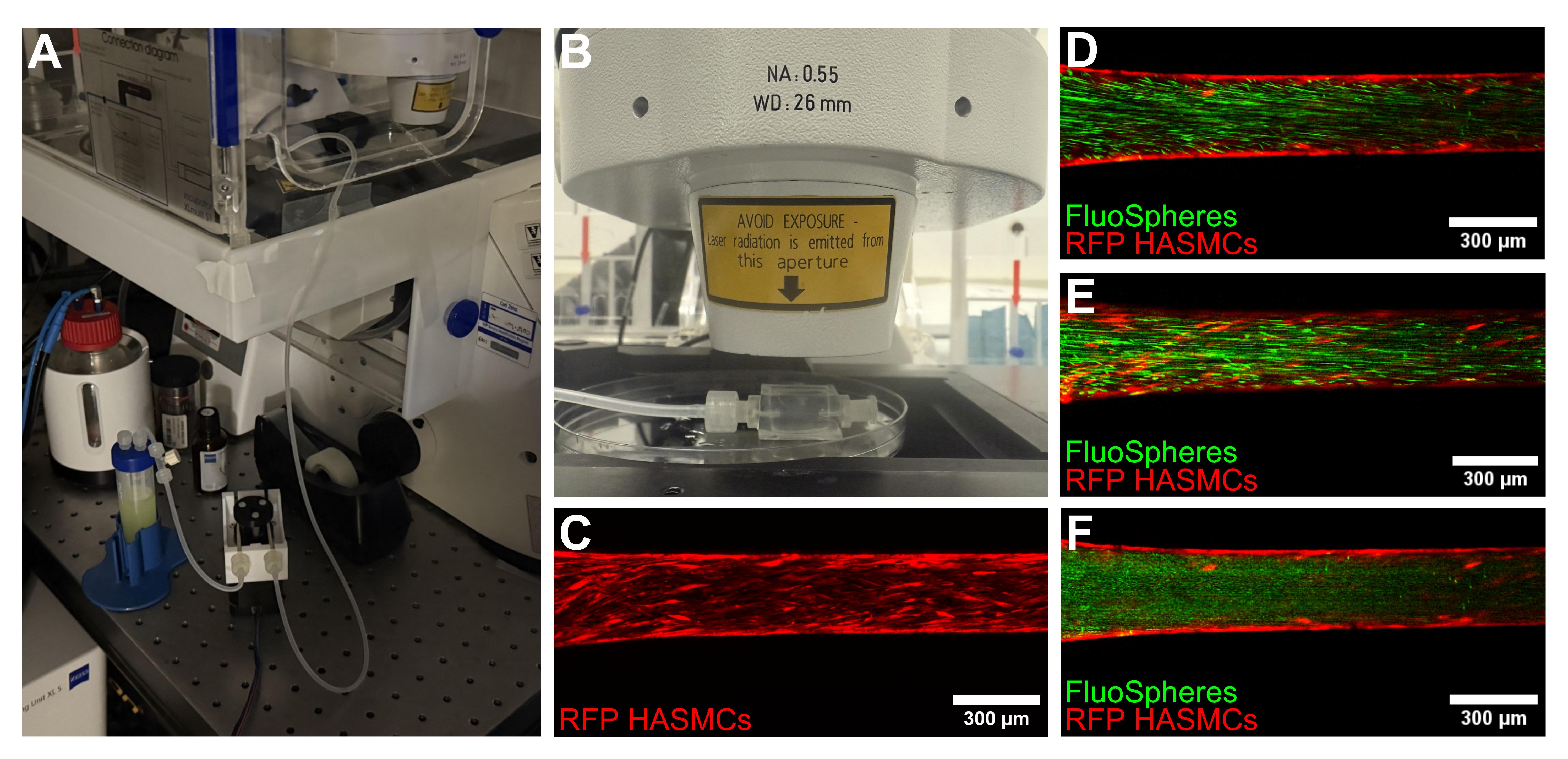
**Fig. S1.** Perfusion of fixed, cell-lined microchannels. **(A)** Perfusion setup with reservoir, peristaltic pump, and tubing. **(B)** Hydrogel device attached to tubing via a Luer connector. **(C)** Flattened z-stack of RFP HASMCs lining channel, prior to perfusion. **(D-F)** Green fluorescent beads flowing through the channel at 5 µL min^-1^, 50 µL min^-1^, and 500 µL min^-1^, respectively.

Figure S2 shows Scanning Electron Microscopy (SEM) imaging of the interior surface of a microchannel and the filament used to pattern it. Neither show topographic features that could drive the SMC alignment seen in our platform


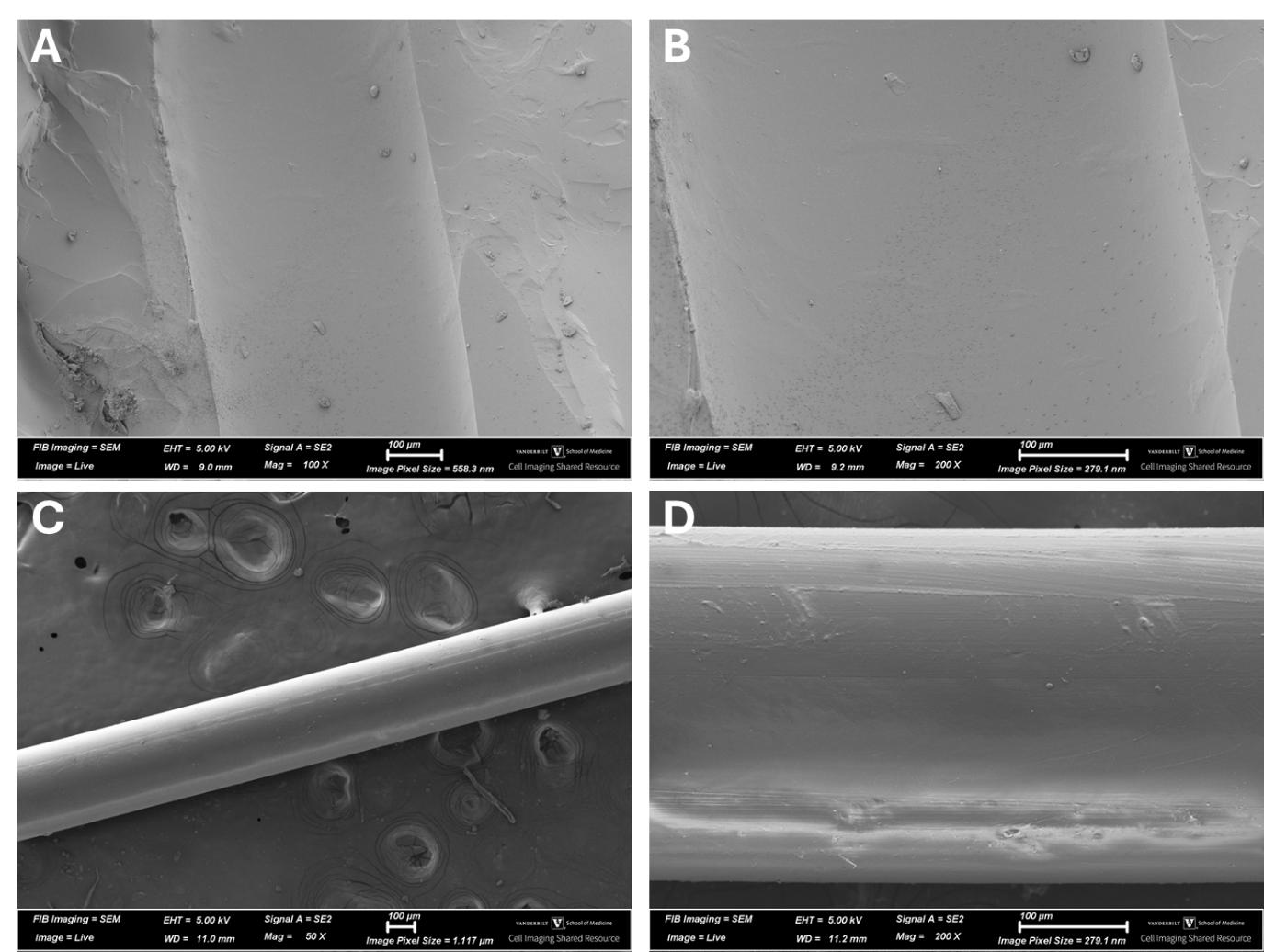


**Fig. S2.** SEM imaging: (Top) Topography of the inner surface of a channel shown at **(A)** 100x and **(B)** at 200x. (Bottom) Nylon filament used to pattern channels at **(C)** 50x and **(D)** at 200x.

Figure S3 illustrates the 3D-printed resin collars designed to compensate for gelatin shrinkage after channel formation and during extended culture immersed in cell culture medium. The barbs were printed with an extended shaft to accommodate adjustments. Initially, the collars were positioned on the outside of the PDMS mold during intital gelatin casting and initial cell introduction. Following gelatin shrinkage, the collars were repositioned onto the shaft on the interior of the PDMS mold to ensure continued contact between the barb and the gelatin and microchannel, allowing for subsequent rounds of cell introduction. STL files for both barbs and collars were modeled in Fusion 360 (Autodesk), sliced using Chitubox, and they were printed on a Phrozen Sonic Mini 8K S resin printer with Liqcreate Bio-Med Clear resin. Collars of various lengths were fabricated to account for varying degrees of gel shrinkage.


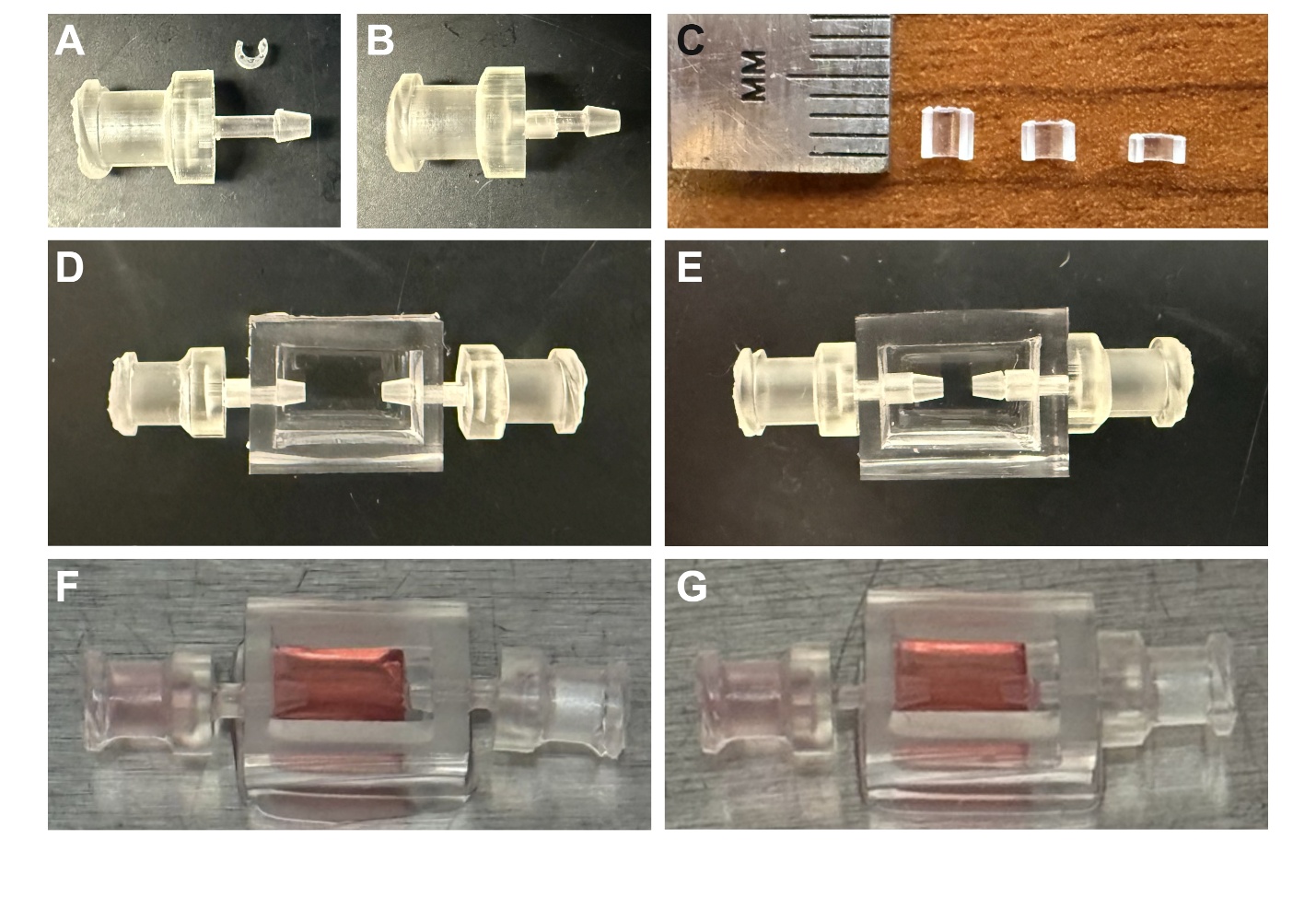


**Fig. S3. (A)** Barb and collar before assembly. (**B)** Barb + collar assembled (**C)** Collars of lengths 2mm, 1.5mm, and 1mm. (**D)** Barb + collar + PDMS mold with the collars on the outside of the mold. (**E)** Barb + collar + PDMS mold with the collars on the inside of the mold.

**Supplemental Video 1** shows a channel perfused with green Fluosphere-spiked PBS at 5 µL min^-1^. Frame time is 1.56 seconds.

**Supplemental Video 2** shows a channel perfused with green Fluosphere-spiked PBS at 50 µL min^-1^. Frame time is 1.56 seconds.

**Supplemental Video 3** shows a channel perfused with green Fluosphere-spiked PBS at 500 µL min^-1^. Frame time is 1.56 seconds. Midway through the video, an air bubble traverses the channel, causing a temporary gap in the fluorescent bead solution.
